# Supplementary material for: Safety and efficacy of combined treatment with tumor-infiltrating lymphocytes and oncolytic adenovirus TILT-123 in metastatic melanoma
Source: Cell Rep Med. 2025 Mar 18;6(3):102016. doi: 10.1016/j.xcrm.2025.102016 (PMC11970381; doi:10.1016/j.xcrm.2025.102016)
Supplement: Document S1. Figures S1–S8 and Tables S1–S4 [file mmc1.pdf]

Cell Reports Medicine, Volume *6*

Supplemental information

Safety and efficacy of combined treatment with tumor-infiltrating lymphocytes and oncolytic adenovirus TILT-123 in metastatic melanoma

Tine J. Monberg, Santeri A. Pakola, Benedetta Albieri, Eva Ellebaek, Marco Donia, Rikke L. Eefsen, Troels H. Borch, Tatiana V. Kudling, Torben Lorentzen, Helle W. Hendel, Cecilie Vestergaard, Cathrine Lorentzen, Rikke B. Holmstroem, Victor Arias, Amir Khammari, Claudia Kistler, João M. Santos, James H.A. Clubb, Lyna Haybout, Marie C.W. Westergaard, Özcan Met, Dafne C.A. Quixabeira, Elise Jirovec, Riikka Havunen, Suvi Sorsa, Victor Cervera-Carrascon, Brigitte Dreno, Akseli Hemminki, and Inge Marie Svane

TILT-T215 Supplementary Material

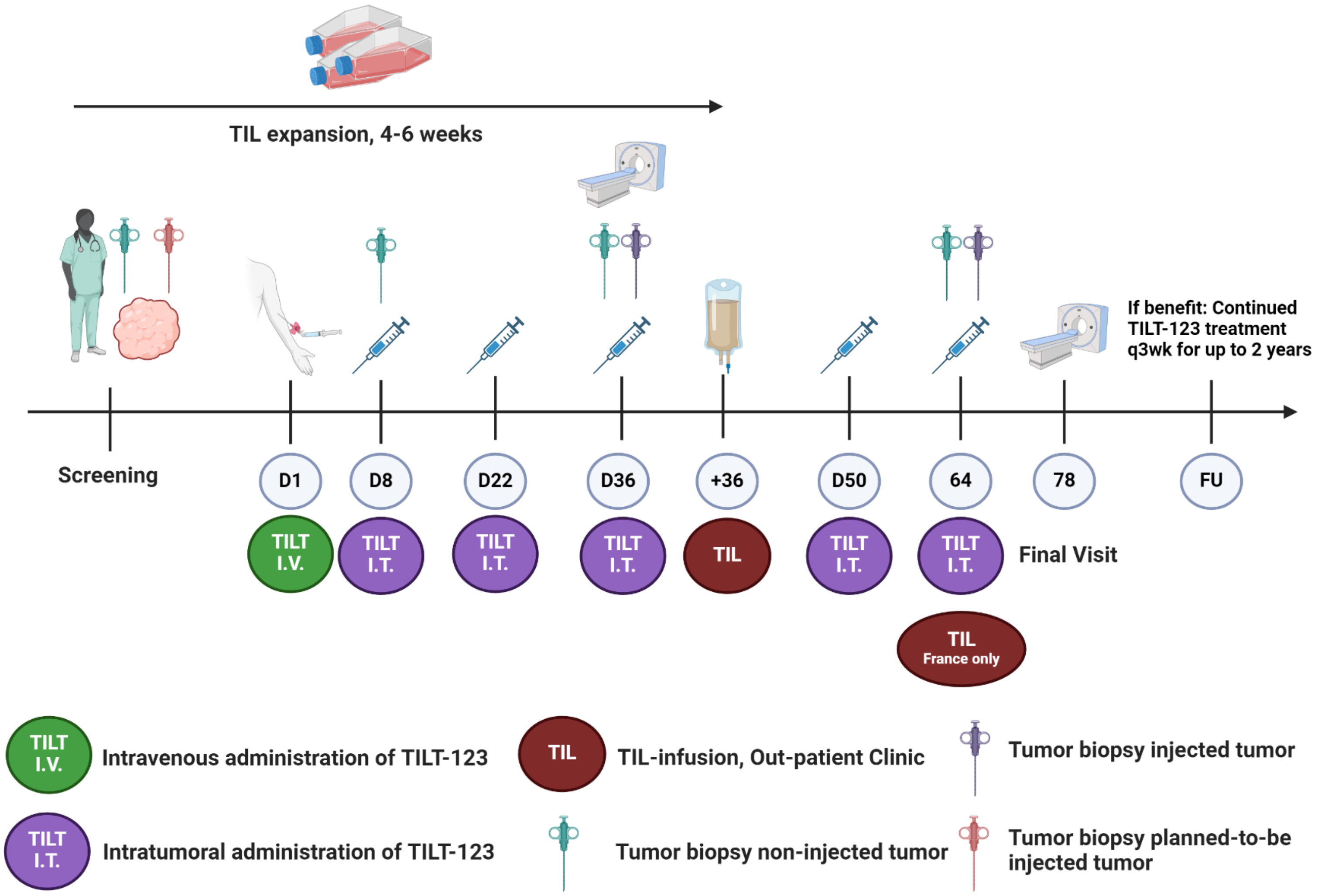

Figure S1: Treatment schedule in TILT-T215. *Tumor tissue for TIL production was resected during the screening and TILs were administered on day 37 or later. Patients received a total of six TILT-123 injections, four before TIL infusion and two post TIL-infusion. If patients benefitted from the treatment, they could continue injections with TILT-123 every third week for up to two years. Related to STAR*METHOD.*

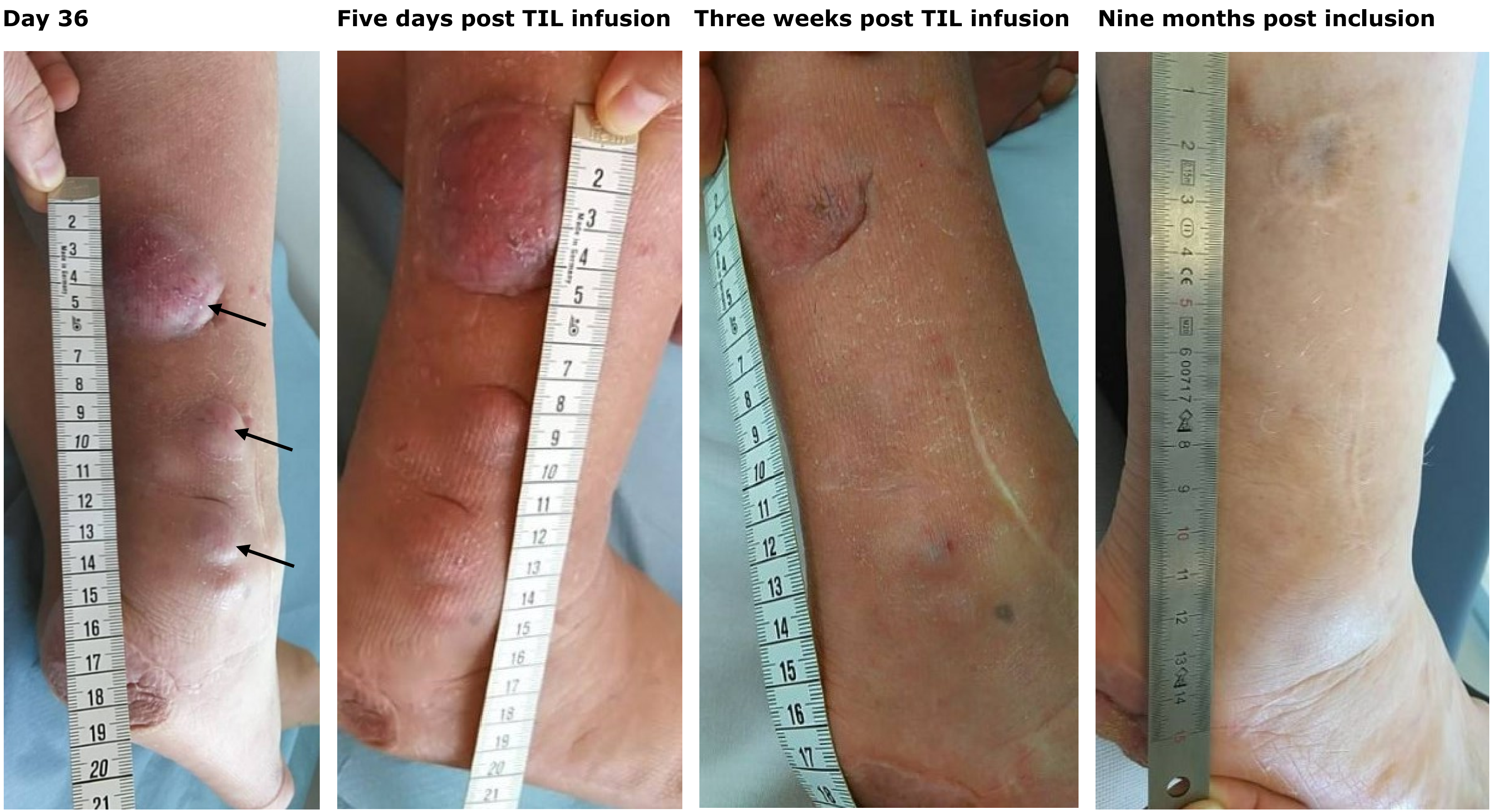

Figure S2: Development of response in patient 101.15. *Patient 101.15 had several subcutaneous metastases on the right lower leg. Before D36 TILT-123 was injected in the three arrow-marked tumors (at least once per tumor). On D36 all subcutaneous metastases had increased in size and the patient experienced increasing pain. Five days after TIL infusion no objective change was observed, but three weeks post TIL infusion all metastases showed a remarkable reduction in size. Nine months post treatment most of these subcutaneous lesions could no longer be visualized. Related to Figure 2.*

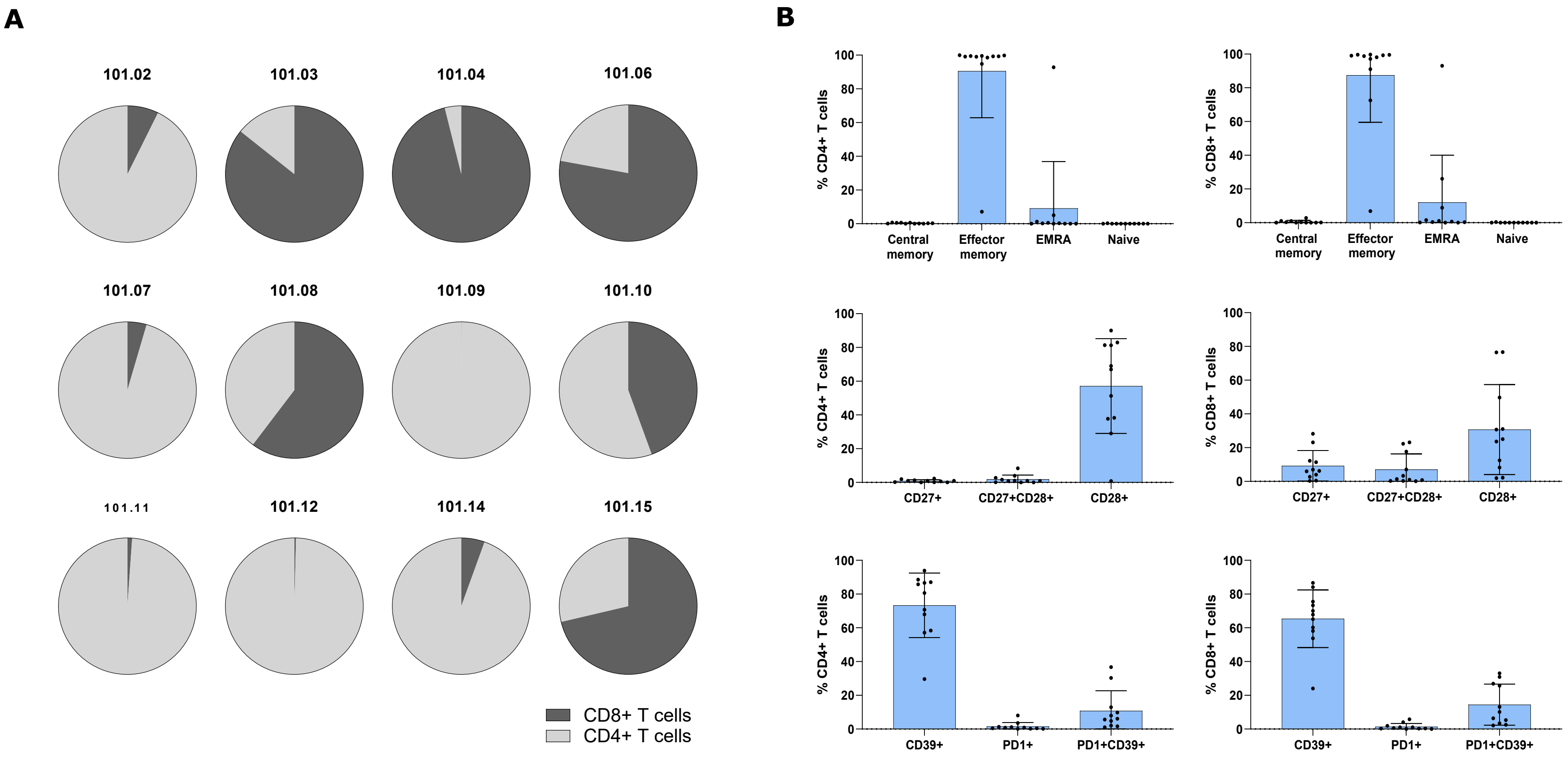

Figure S3: Phenotyping of TIL infusion products. (A) *Distribution of CD4+ and CD8+ T cells out of the living cells in the infusion product from 12 patients treated at the Danish site.* ***(B)*** *Phenotypic characterization of the TIL infusion products from 12 Danish patients. Data are presented as mean ± standard deviation (SD). Related to STAR*METHOD*

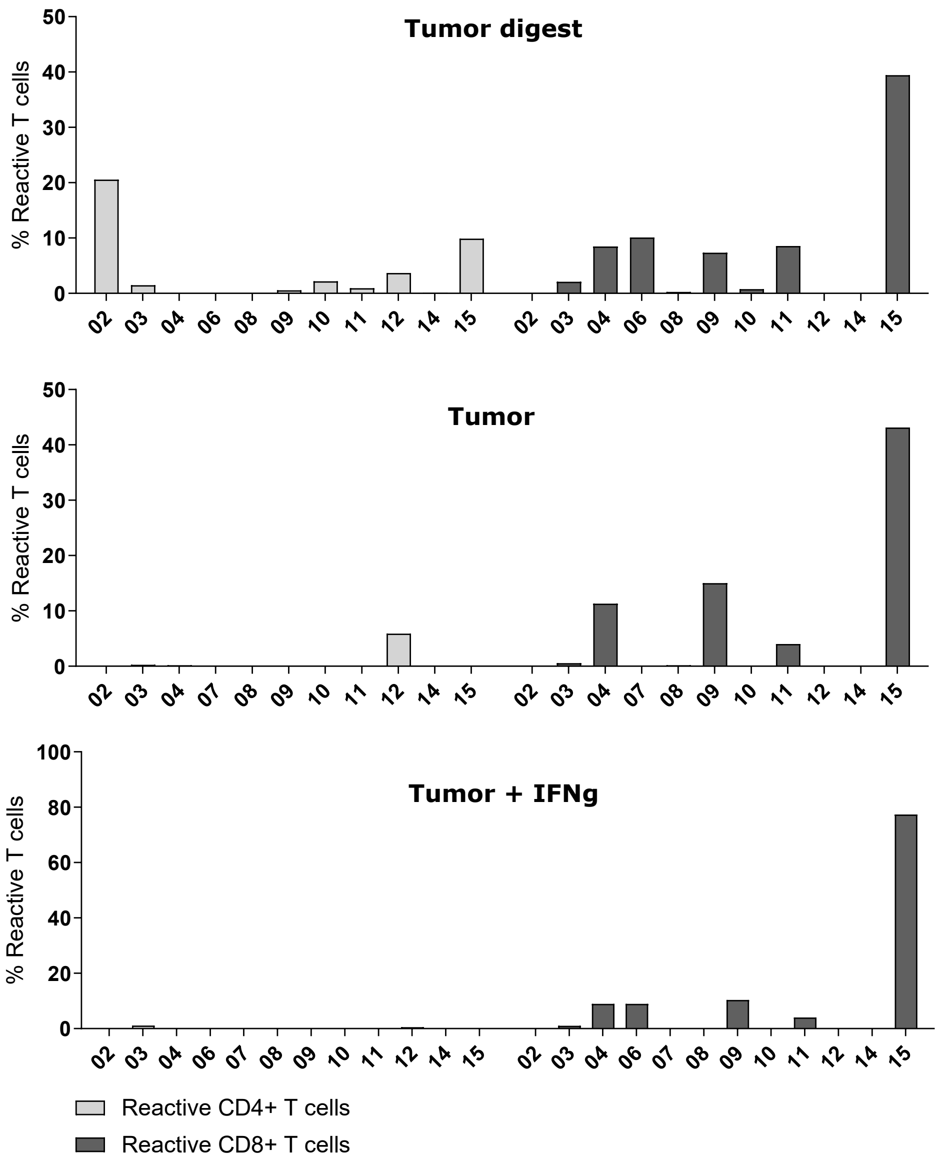

Figure S4: Assessment of tumor reactivity. *Infusion product reactivity towards autologous tumor digest, tumor cells and tumor cells pre-treated with IFNg was performed using multicytokine intracellular staining. Reactive cells were simultaneously positive to two out of the four reactivity markers used (CD137, CD107a, TNF, IFNg). Related to Figure 2.*

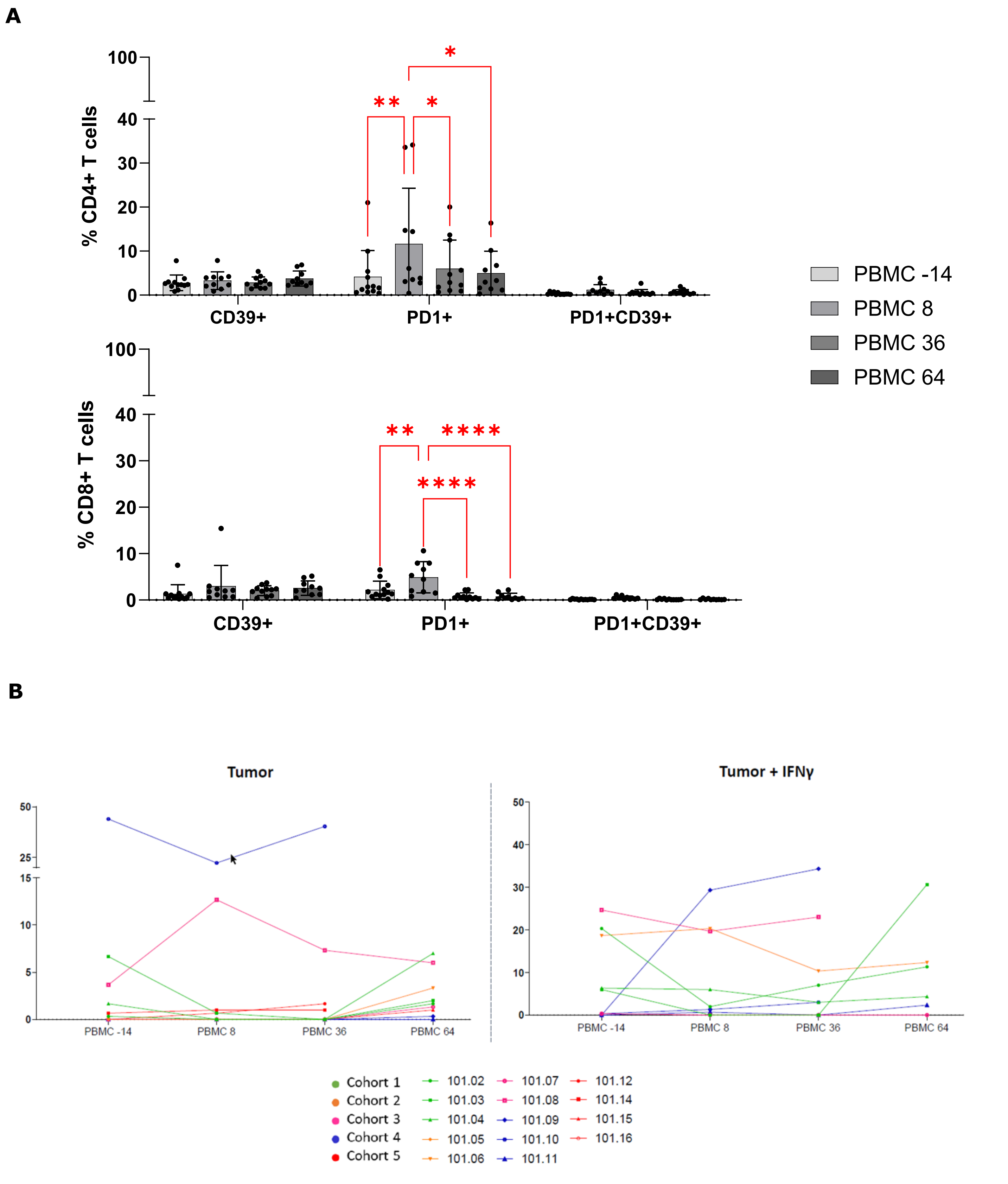

Figure S5: Phenotype and reactivity of PBMCs*. (A) Phenotypic characterization of PBMCS isolated from whole blood at different timepoints of the study. Significantly higher expression of PD1 on day 8 was observed in both peripheral CD4+ and CD8+ T cells populations. PBMCs phenotyping was performed on samples from patients treated at the Danish site (n=13). Data are presented as mean ± standard error of the mean (SEM). Statistical significance was evaluated using a two-way ANOVA followed by Tukey's multiple comparison test and indicated as p* <0.05, p** <0.01, p***<0.001 and p****<0.0001.* ***(B)*** *PBMCs reactivity towards tumor cells (with or without IFNg pre-treatment) was assessed using ex vivo IFNg ELISpot. Related to STAR*METHOD.*

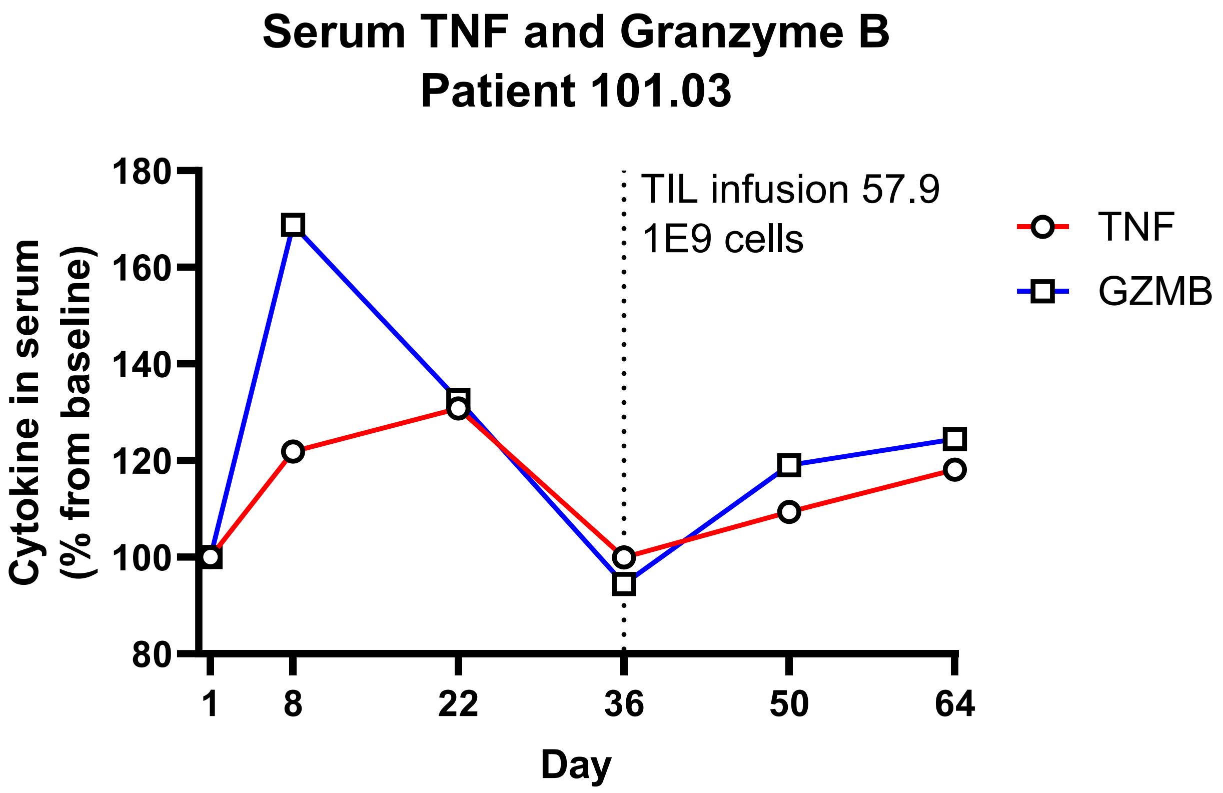

Figure S6: Pre-treatment serum cytokine levels of patient 101-03 on days 1, 8, 22, 36, 50 and 64. Related to STAR*METHOD

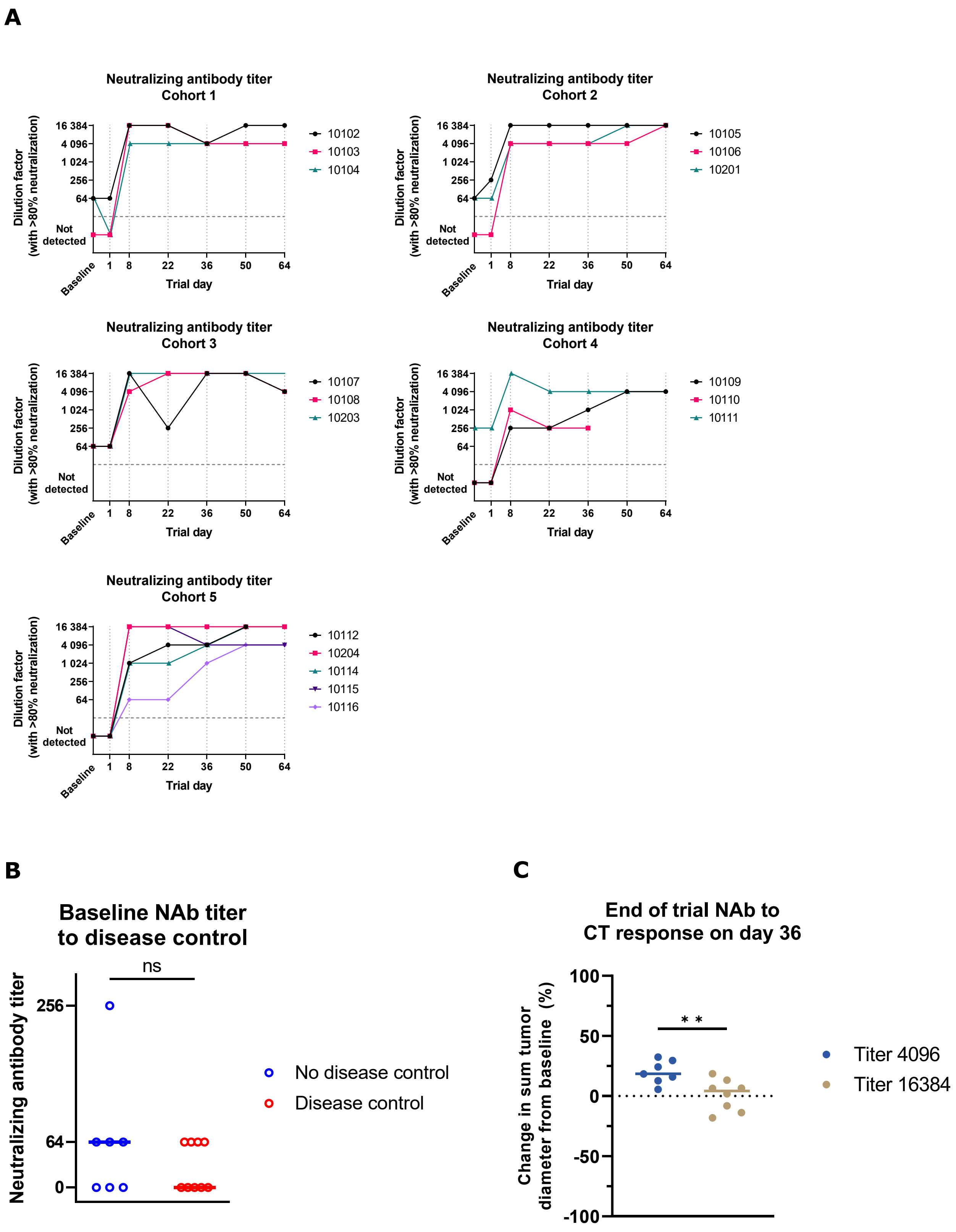

Figure S7: NAB titer measurement. *(A) Neutralizing antibody titer against TILT-123 across trial in all cohorts.* ***(B)*** *Baseline neutralizing antibody titer against TILT-123 in patients with or without disease control assessed with RECIST 1.1. at the end of the trial, n=16. ns=non-significant* ***(C)*** *End of trial neutralizing antibody titer against CT response on day 36. Groups compared with Mann-Whitney U-test. The mean value is indicated by the horizontal line. Statistical significance is indicated as p** <0.01. Related to STAR*METHOD*

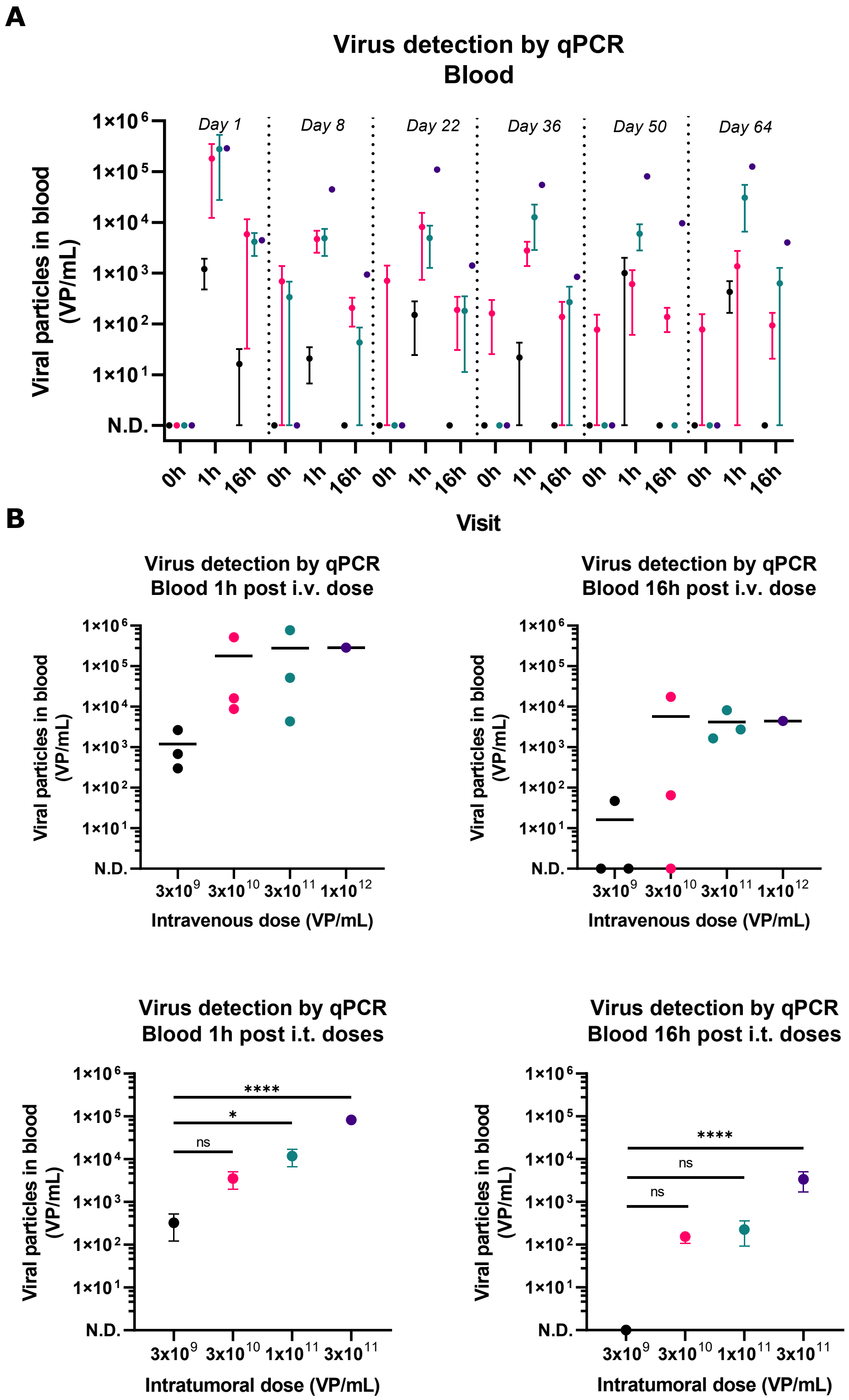

Figure S8: Virus genome detection in blood. ***(A)*** *Virus genome detection from blood across trial from cohorts 1-4.* ***(B)*** *Virus genome detection from blood 1 hour and 16 hours after intravenous (i.v.) and intratumoral (i.t.) doses. Groups were compared using two-way ANOVA followed by Fisher's Least Significant Difference (LSD) test. Mean values are indicated with horizontal lines for i.v. dose groups. Data is presented as mean ±* standard error of the mean (*SEM*) for i.t. dose groups. *Statistical significance is indicated as p* <0.05 and p****<0.0001. ns= non-significant. Related to STAR*METHOD.*

**Patient**	**Localization of tumor resected for TIL expansion**	**Number of cells in TIL infusion product (x 10^9)**	**Localization of injected tumor(s)**
**101.02**	Adrenal gland	36.8	Liver metastases
**101.03**	Subcutaneous	57.9	Subcutaneous tumors on leg
**101.04**	Lymph node	65.4	Tumors in left axillar
**101.05**	Liver	Na	Liver metastases
**101.06**	Subcutaneous	86.8	Liver metastases
**101.07**	Lymph node	69.2	Lymph node on neck
**101.08**	Subcutaneous	47.6	Lymph nodes on thoracic wall and neck
**101.09**	Subcutaneous	62.4	Liver tumor and subcutaneous tumor
**101.10**	Lymph node	63.2	Subcutaneous tumor on thoracic wall
**101.11**	Lymph node	17.9	Lymph nodes/inguinal tumors
**101.12**	Intraabdominal	28.7	Liver metastases
**101.14**	Lung	32.7	Liver metastases
**101.15**	Lymph node	44	Subcutaneous tumors on leg, Lymph node in inguen
**101.16**	Subcutaneous	48.9	Subcutaneous tumors on breast
**102.01**	Lymph node	2.58 + 4.9	Palate tumor<br>Tumor under right scapula<br>Tumors on left thigh and right thigh
**102.03**	Subcutaneous	3.51	Right axillary tumor, subcutaneous tumors on arm
**102.04**	Lymph node	3 + 2.1	Lymph node in inguen

Table S1: Treatment characteristics. *Localizations of resected tumors for TIL expansion, number of infused cells in the TIL product and localization of tumors injected with TIL-123. Related to STAR*METHOD.*

	Grade 1			Grade 2			Grade 3			Grade 4			Total	Unique cases
**Treatment Related Adverse Event**	**TILT-123**	**TIL**	**Both**	**TILT-123**	**TIL**	**Both**	**TILT-123**	**TIL**	**Both**	**TILT-123**	**TIL**	**Both**		
**General symptoms**														
Anorexia	1			1									2	2/17
Arthralgia			1										1	1/17
Asthenia	1		1	1									3	1/17
Dizziness	1												1	1/17
Fatigue	3	1	2										6	5/17
Fatigue worsening				1									1	2/17
Headache	3	1											4	3/17
Worsening of headache				3									3	1/17
Hypotension					1								1	1/17
Dry skin	1					1							2	2/17
Memory impairment			1										1	1/17
Flushing	1		1										1	2/17
Myalgia	3												3	2/17
Pain	1												1	1/17
Severe myodesopsia at rest	1												1	1/17
Maculopapular rash	2					1							2	3/17
**Local symptoms**														
Alopecia	2		1										3	3/17
Bilateral edema						1							1	1/17
Diarrhea	3												3	2/17
Edema, by the left eye	1												1	1/17
Lacrimation	2												2	1/17
Left thorax pain					1								1	1/17
Pain left knee	1												1	1/17
Pain left upper extremity	1												1	1/17
Pain liver			1										1	1/17
Pain right leg				1									1	1/17
Pruritus right crus	1												1	1/17
Muscle pain right calf	1												1	1/17
Skin redness right crus	1												1	1/17

Skin redness right groin	1												1	1/17
Swollen and tender lymph nodes right neck	1					1							2	1/17
**Infectious symptoms**														
Febrile syndrome		1											1	1/17
Fever	11	1	1	4	4	1	2	1					25	13/17
Chills	2		2		3	1		1					9	7/17
Abscess right side of thorax				1									1	1/17
**Injection related symptoms**														
Infection injection site				1									1	1/17
Itching at the injection sites	1												1	1/17
Injection site pain	3						1						4	3/17
**Hematology/lab values**														
Increased ALAT		1											1	1/17
Increased ASAT								1					1	1/17
Increased INR					1								1	1/17
Lymphopenia	1												1	1/17
TSH decreased	1												1	1/17
Prolonged QTcB interval			1										1	1/17
**Gastrointestinal**														
Nausea	4	2		2									8	6/17
Oral mucositis	1												1	1/17
Vomiting	2				1								3	2/17
**Other**														
Hypoxia					1								1	1/17
TIL reaction								1					1	1/17
Worsening of adrenal insuffciency											1			1/17
Vitiligo	2												2	2/17

Table S2: Adverse events. *Frequency and registered CTCAE grade of all treatment related adverse events according to CTCAE v.5.0. Related to Table 2.*

Patient	Gender	Histology	Treatment Cohort	Response day 36			BOR day 78 or later		
				RECIST 1.1	iRECIST	PET	RECIST 1.1	iRECIST	PET
**101.02**	Male	Mucosal	1	SD	iSD	SMD	PD	iUPD	PMD
**101.03**	Female	Cutaneous	1	PD	iUPD	PMD	SD	iSD	PMD
**101.04**	Male	Cutaneous	1	PD	iUPD	PMD	PD	iCPD	PMD
**101.05**	Male	Uveal	2	SD	iSD	NA	SD	iSD	NA
**101.06**	Male	Uveal	2	PD	iUPD	PMD	PD	iCPD	PMD
**102.01**	Female	Mucosal	2	PD	iUPD	SMD	PD	iCPD	PMD
**101.07**	Female	Mucosal	3	SD	iSD	PMR	CR*	iCR*	PCR*
**101.08**	Male	Cutaneous	3	PD	iUPD	PMD	PD	iCPD	PMD
**102.03**	Male	Cutaneous	3	PD	iUPD	SMD	PD	iCPD	SMD
**101.09**	Female	Uveal	4	PD	iUPD	SMD	PD	iCPD	SMD
**101.10**	Female	Mucosal	4	SD	iSD	MMR	SD	iSD	MMR
**101.11**	Female	Cutaneous	4	PD	iUPD	PMD	PD	iCPD	SMD
**101.12**	Female	Mucosal	5	PD	iUPD	PMD	NA	NA	NA
**101.14**	Male	Uveal	5	SD	iSD	PMR	SD	iSD	PMR
**101.15**	Female	Cutaneous	5	PD	iUPD	MMR	PR	iPR	PMR
**101.16**	Female	Cutaneous	5	PD	iUPD	SMD	PD	iCPD	PMD
**102.04**	Female	Cutaneous	5	SD	iSD	MMR	PD	iUPD	PMD

Table S3: Responses evaluated by RECIST 1.1, iRECIST and PET. *TILT-123 treatment alone was evaluated on day 36 before the infusion of TILs. TILT-123 and TIL treatment was evaluated as best overall response (BOR) on day 78 or later for patients continuing in the extension phase of the trial. * Pathological confirmed complete response. Patient 101.05 had PET-negative lesions at baseline. Patient 101.12 died from cancer progression before D78 evaluation. Related to Figure 2 and Figure 5.*

	Target	Fluorochrome	Clone	Species	specificity	Catalog number	Distributor
Golgi solution	CD107a	BV421	H4A3	mouse	anti-human	562623	BD
	GolgiStop™	Protein transport inhibitor containing Monensin				554724	BD
	GolgiPlug™	Protein transport inhibitor containing Brefeldin A				555029	BD
Extracellular staining ICS	CD4	BV711	SK3	mouse	anti-human	563028	BD
	CD8	QD605	3B5	mouse	anti-human	Q10009	Invitrogen
	CD56	BV510	NCAM16.2	mouse	anti-human	563041	BD
	CD3	PE-CF594	UCHT1	mouse	anti-human	562280	BD
Intracellular staining ICS	TNFα	APC	MAb11	mouse	anti-human	554514	BD
	IFN-γ	PeCy7	B27	mouse	anti-human	557643	BD
	CD137	PE	4B4-1	mouse	anti-human	555956	BD
Phenotyping panel 1	CD3	BV786	SK7	mouse	anti-human	563800	BD
	HLA-DR	PerCP-Cy5.5	G46-6	mouse	anti-human	560652	BD
	CCR7	PE	G043H7	mouse	anti-human	353204	Nordic biosite
	CD8	APC-R700	RPA-T8	mouse	anti-human	565165	BD
	CD39	BV421	TU66	mouse	anti-human	563679	BD
	CD4	BV510	SK3	mouse	anti-human	562970	BD
	CD56	BV605	NCAM16.2	mouse	anti-human	562780	BD
	CD45RA	BV650	HI100	mouse	anti-human	563963	BD
	PD-1	PE/Dazzle 594	EH12.2H7	mouse	anti-human	329940	Nordic biosite
	CD38	PE-CY5	HIT2	mouse	anti-human	555461	BD
Phenotyping panel 2	CD127	FITC	REA614	mouse	anti-human	130-113-417	Miltenyi Biotec
	CXCR3	BB700	1C6	mouse	anti-human	566532	BD
	CCR4	APC	L291H4	mouse	anti-human	359408	Nordic biosite
	CCR6	BV711	11A9	mouse	anti-human	563923	BD
	CD3	BV786	SK7	mouse	anti-human	563800	BD
	CD4	BV510	SK3	mouse	anti-human	562970	BD
	CD56	BV605	NCAM16.2	mouse	anti-human	562780	BD
	CD8	APC-R700	RPA-T8	mouse	anti-human	565165	BD
	CD25	PE-CY7	2A3	mouse	anti-human	335824	BD
Phenotyping panel 3	CD3	BV786	SK7	mouse	anti-human	563800	BD
	CD19	BV711	SJ25C1	mouse	anti-human	563036	BD
	HLA-DR	PerCP-Cy5.5	G46-6	mouse	anti-human	560652	BD
	CD1c	APC	L161	mouse	anti-human	331524	Nordic biosite
	CD33	BV510	WM53	mouse	anti-human	563257	BD
	CD123	BV605	7G3	mouse	anti-human	564197	BD
	CD11c	BV650	B-ly6	mouse	anti-human	563404	BD
	CD56	BV605	NCAM16.2	mouse	anti-human	562780	BD
	CD141	PE	1A4	mouse	anti-human	559781	BD
	CD16	PE/AF700	3G8	mouse	anti-human	101611P1	AAT Bioquest
	CD14	PE-CFE594	MϕP9	mouse	anti-human	562335	BD

Table S4: List of antibodies used for multicytokine intracellular staining and phenotyping. *Related to STAR*METHOD*
