# Supplementary material for: Safety and efficacy of combined treatment with tumor-infiltrating lymphocytes and oncolytic adenovirus TILT-123 in metastatic melanoma
Source: Cell Rep Med. 2025 Mar 18;6(3):102016. doi: 10.1016/j.xcrm.2025.102016 (PMC11970381; doi:10.1016/j.xcrm.2025.102016)
Supplement: Document S2. Article plus supplemental information [file mmc2.pdf]

Cell Reports
Medicine

Article

Safety and efficacy of combined treatment with tumor-infiltrating lymphocytes and oncolytic adenovirus TILT-123 in metastatic melanoma

Graphical abstract

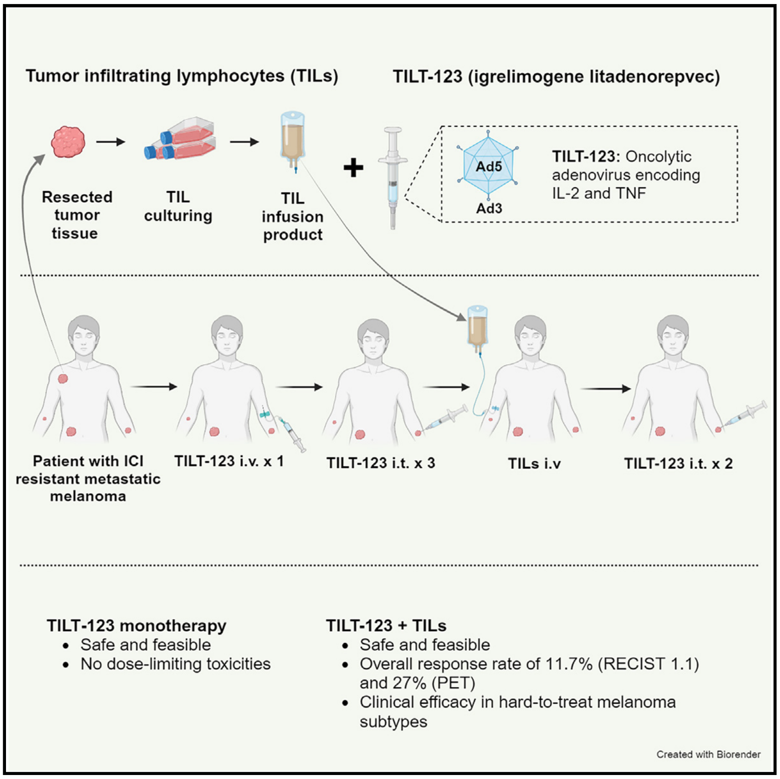

Highlights

- Oncolytic adenovirus TILT-123 monotherapy is safe and feasible in patients with MM
- Combined treatment with TILT-123 and TILs has low toxicity
- TILT-123 + TILs can result in durable responses in patients with ICI-resistant MM

Authors

Tine J. Monberg, Santeri A. Pakola, Benedetta Albieri, ..., Brigitte Dreno, Akseli Hemminki, Inge Marie Svane

Correspondence

akseli.hemminki@helsinki.fi (A.H.),
inge.marie.svane@regionh.dk (I.M.S.)

In brief

Monberg et al. demonstrate that the oncolytic adenovirus TILT-123, alone or in combination with tumor-infiltrating lymphocytes (TILs), can be safely administered to patients with immune checkpoint inhibitor (ICI)-resistant metastatic melanoma (MM). The combined treatment results in durable objective responses, including a complete response in a patient with mucosal melanoma.

Monberg et al., 2025, Cell Reports Medicine 6, 102016
March 18, 2025 © 2025 The Authors. Published by Elsevier Inc.
https://doi.org/10.1016/j.xcrm.2025.102016

CellPress

Cell Reports Medicine

CellPress
OPEN ACCESS

Article

Safety and efficacy of combined treatment with tumor-infiltrating lymphocytes and oncolytic adenovirus TILT-123 in metastatic melanoma

Tine J. Monberg,[1,9] Santeri A. Pakola,[2,9] Benedetta Albieri,[1] Eva Ellebaek,[1] Marco Donia,[1] Rikke L. Eefsen,[1] Troels H. Borch,[1] Tatiana V. Kudling,[2] Torben Lorentzen,[3] Helle W. Hendel,[4] Cecilie Vestergaard,[1] Cathrine Lorentzen,[1] Rikke B. Holmstroem,[1] Victor Arias,[2] Amir Khammari,[5] Claudia Kistler,[6] João M. Santos,[2,6] James H.A. Clubb,[2,6] Lyna Haybout,[2,6] Marie C.W. Westergaard,[1] Özcan Met,[1,7] Dafne C.A. Quixabeira,[2,6] Elise Jirovec,[2] Riikka Havunen,[2,6] Suvi Sorsa,[6] Victor Cervera-Carrascon,[2,6] Brigitte Dreno,[5] Akseli Hemminki,[2,6,8,11,10,*] and Inge Marie Svane[1,10,*]

[1]National Center for Cancer Immune Therapy (CCIT-DK), Department of Oncology, Copenhagen University Hospital, 2730 Herlev, Denmark
[2]Cancer Gene Therapy Group, Translational Immunology Research Progam, University of Helsinki, Helsinki, Finland
[3]Department of Gastroenterology, Copenhagen University Hospital, 2730 Herlev, Denmark
[4]Department of Clinical Physiology and Nuclear Medicine, Copenhagen University Hospital, 2730 Herlev, Denmark
[5]Nantes Université, INSERM, CNRS, Immunology and New Concepts in ImmunoTherapy, INCIT, UMR 1302/EMR6001, 44000 Nantes, France
[6]TILT Biotherapeutics Ltd, Helsinki, Finland
[7]Department of Health Technology, Technical University of Denmark, 2800 Lyngby, Denmark
[8]Helsinki University Hospital Comprehensive Cancer Center, Helsinki, Finland
[9]These authors contributed equally
[10]These authors contributed equally
[11]Lead contact
*Correspondence: akseli.hemminki@helsinki.fi (A.H.), inge.marie.svane@regionh.dk (I.M.S.)
https://doi.org/10.1016/j.xcrm.2025.102016

SUMMARY

Tumor-infiltrating lymphocytes (TILs) are effective in the treatment of metastatic melanoma (MM), but toxicity limits its application. TILT-123 (igrelimogene litadenorepvec) is an oncolytic adenovirus producing interleukin-2 (IL-2) and tumor necrosis factor (TNF) upon replication. In this phase 1 trial, 17 patients with metastatic checkpoint inhibitor-resistant melanoma are treated with TILT-123 and TILs without preconditioning chemotherapy or postconditioning IL-2. The treatment is safe and feasible. According to computed tomography (CT), the objective response rate is 11.7% (2/17) and disease control is observed in 35% (6/17), including a partial response lasting >8 months and a durable complete response in a mucosal melanoma patient. According to positron emission tomography (PET), disease control is observed in 7/15 (47%) with minor or partial responses in 4/15 (27%). In the initial TILT-123 monotherapy phase of the trial, disease control is observed in 6/17 (35%) and 10/16 (63%) in CT and PET, respectively. The study demonstrates good tolerability and preliminary efficacy.

INTRODUCTION

Tumor-infiltrating lymphocyte (TIL) therapy was recently approved by the United States Food and Drug Administration (FDA) for patients with unresectable or metastatic melanoma (MM)-resistant to PD-1 inhibition.[1] This approval was a promising step toward a broader access to TIL therapy, but, still, regulatory and practical challenges limit the implementation of the treatment. Further, strategies to reduce the toxicity of TIL therapy while maintaining its effectiveness are needed to extend the treatment to more patients. Several ongoing studies aim to increase the efficacy of TIL therapy by combining it with other types of immunotherapies. However, the ideal companion for TIL therapy is to be found.

Oncolytic virus (OV) therapy is a promising candidate for combination with TILs, as the two approaches may work synergistically. OVs infect and kill cancer cells resulting in lysis of the tumor cells and thereby release of tumor antigens. This, along with the natural pro-inflammatory response from the viral vector, stimulates immune cells and elicits an antitumor response. During the recent years, increasing attention has been directed toward the immune stimulatory properties of OVs rather than their direct oncolytic effect.[2] To date, only one OV therapy, the herpes simplex virus (HSV) talimogene laherparepvec (T-VEC), has been approved by the FDA and EMA (European Medicines Agency) for the treatment of MM. Nonetheless, the family of OVs is diverse,[2] and choosing the one(s) with the greatest T cell stimulatory potential is presumably the way to overcome T cell therapy limitations.[3] Thus, there is a need for further trials to understand which combinations of OVs and immunotherapies are most effective. The combination of immune checkpoint inhibitors (ICIs) and OV was tested in the phase 1b/3 MASTERKEY-265 trial (ClinicalTrials.gov identifier: NCT02263508).[4] In the phase

This is an open access article under the CC BY license (http://creativecommons.org/licenses/by/4.0/).

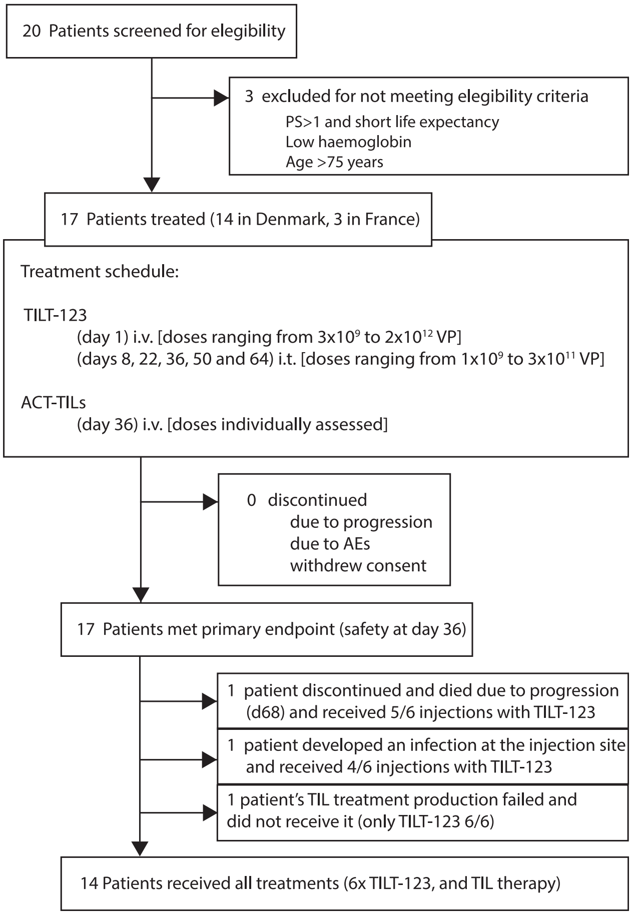

Figure 1. TILT-T215 CONSORT diagram

1b part of this study, a combined treatment with T-VEC and pembrolizumab in patients with MM showed a high complete response (CR) rate of 43%. However, in the later phase 3 trial, no benefit on progression-free survival (PFS) or overall survival (OS) was reported when comparing T-VEC and pembrolizumab with placebo and pembrolizumab. Possible explanations for the failure of MASTERKEY-265 were the inclusion of patients with more advanced disease and larger tumor lesions compared to the phase 1 study.[5] HSV agents are known to work better when injected into smaller tumors and in patients with limited disease burden[6]; thus, these differences in the patient population might be of great importance. Also, differences in the timing of the virus injection in relation to the ICI treatment might have influenced efficacy.[5] Indeed, the sequence of these therapies can influence response,[7] and there is a lack of knowledge on whether OV therapy should be administered before, after, or concomitantly with different types of immunotherapy.

Overall, a better understanding of the relation between OV therapy and other immune therapeutic agents is needed, but, in the context of TIL therapy, preclinical data suggest that oncolytic adenovirus might be the most optimal partner.[8,9]

TILT-123 is an oncolytic serotype 5/3 chimeric adenovirus modified with a 24 kb deletion in the E1A region and the addition of an E2F promoter upstream of E1A. These modifications enable the virus to selectively replicate in cancer cells with defects in the p16/retinoblastoma/E2F pathway. In conjunction with replication, the virus produces interleukin-2 (IL-2) and tumor necrosis factor (TNF), which results in the recruitment of immune cells to the tumor microenvironment (TME). Further, tumor cells infected by the virus will undergo lysis, inducing tumor antigen spread in the TME. In a preclinical murine setting, combining an adenovirus encoding IL-2 and TNF with TILs showed increased antitumor efficacy in comparison to single-agent treatment with virus therapy or TIL therapy in melanoma.[10] These findings were supported in an animal model in which tumor growth in Syrian hamsters was reduced significantly when treated with a combination of TILs and IL-2/TNF-coding oncolytic adenovirus, compared to single-agent therapy.[11] Additionally, preclinical data show that combining OV therapy and TIL therapy can omit the need for preconditioning chemotherapy and postconditioning IL-2.[12,13]

TILT-123 monotherapy was recently administered to patients with different solid cancers in the phase 1 trial TUNIMO (ClinicalTrials.gov identifier: NCT04695327).[14] The treatment was safe and feasible with no dose-limiting toxicities (DLTs). Encouragingly, objective responses were also observed.[14]

In this report, we describe the results of the clinical trial TILT-T215 combining oncolytic adenovirus TILT-123 with TIL therapy in patients with ICI-resistant MM. TILT-123 was administered intravenously (i.v.) and intratumorally (i.t.) in a dose-escalating manner in five cohorts while TILs were grown from (pre-TILT-123) resected tumor tissue and administered to the patient in between virus injections without pre- or postconditioning treatment regimens.

The primary endpoint was safety of TILT-123 by day 36, prior to TIL administration. Secondary endpoints included safety and tolerability of the combined treatment and objective response.

RESULTS

Study population

Seventeen patients with MM were included in the trial between February 2020 and November 2023. The trial CONSORT (Consolidated Standards of Reporting Trials) flow diagram is presented in Figure 1. Of the 17 enrolled patients, 14 patients completed therapy by receiving TIL therapy and all six doses of TILT-123. Two patients (101.10 and 101.12) were discontinued from the protocol on day 50 and 61, due to infection at the injection site or rapid disease progression, respectively. Further, one patient (101.05) did not receive TIL therapy due to unsuccessful TIL expansion. This patient completed all virus injections.

Baseline patient characteristics are shown in Table 1. Patients enrolled in the trial had anti-PD-1 or anti-PD-1/anti-CTLA-4-resistant cutaneous (*n* = 8), mucosal (*n* = 5), or uveal (*n* = 4) MM, and most patients were heavily pretreated with a median of 2 (1–7) prior cancer treatment lines. See Figure S1 for the trial schedule.

Feasibility and treatment-related toxicity

Overall, injections with TILT-123 were feasible into various tumor sites, and TILs were successfully expanded from diverse tumor

Table 1. Baseline characteristics of patients enrolled in TILT-T215

Baseline characteristics, n = 17	
Histology: n (%)	
Cutaneous	8 (47%)
Mucosal	5 (29%)
Uveal	4 (24%)
Age in years, median (range)	67 (25–75)
WHO performance status: n (%)	
0	12 (71%)
1	5 (29%)
Gender: n (%)	
Female	10 (59%)
Male	7 (41%)
BRAF: n (%)	
V600E	3 (18%)
Wild type	14 (82%)
Disease stage at entry: n (%)	
IIIc	4 (24%)
IV	13 (76%)
Prior systemic treatment lines for metastatic disease, median (range)	2 (1–7)
PD-1 inhibitor: n (%)	17 (100%)
CTLA4 inhibitor: n (%)	15 (88%)

Table 2. Toxicity evaluated on day 36 after the first three injections of TILT-123

	Registrations	Individual patients (n)	% of patients (CI)
AEs with highest frequency, all CTCAE grades			
Fever	15	9	52.9% (29%–76%)
Nausea	5	4	23.5% (8%–50%)
Fatigue	4	4	23.5% (8%–50%)
Injection site pain	3	2	11.8% (21%–38%)
AEs grade $\geq$ 3			
Fever	1	1	5.9% (0.3%–31%)

locations (Table S1). Adverse events (AEs) were evaluated on day 36 prior to administration of TILs. The most frequently reported AEs related to the treatment with TILT-123 were fever (53%), fatigue (24%), and nausea (24%) (Table 2). Injection site pain was registered in two (12%) of the patients on day 36. One patient experienced grade 3 fever following injection with TILT-123, but the remaining AEs were mild (grade 1–2). Except for one patient with dry skin and one patient with nausea, all AEs experienced before day 36 resolved spontaneously or upon standard management.

The number and types of AEs related to TILT-123 at the end of the study were comparable with those seen before TIL administration. After receiving both therapies, the most frequently reported AEs related to TILT-123 were fever (65%), fatigue (29%), and nausea (29%).

Registered grade 3–4 AEs at the end of the study are presented in Table 3. Overall, only two grade 3 events were registered related to TILT-123 (fever and injection site pain). These AEs resolved within a few days after treatment.

No DLTs were observed in any of the cohorts (for dose escalation, see STAR Methods). However, one patient developed a bacterial infection at the injection site four days after TILT-123 treatment on day 36. The patient received TILs on day 37, but the treatment with TILT-123 was discontinued. The infection resolved upon treatment with antibiotics.

Similarly, TIL therapy was well tolerated with expected side effects such as fever (47%) and chills (35%). However, one patient (101.12) with known adrenal insufficiency had a severe reaction to TIL therapy (grade 3). The patient developed high fever 7 h after TIL infusion, and, despite increased dose of oral hydrocortisone, symptoms of acute adrenal insufficiency (grade 4) later emerged. Symptoms included confusion, hypotension, and dyspnea. The patient was treated with high doses of intravenous hydrocortisone with immediate effect, which allowed complete recovery and hospital discharge within a few days. However, due to rapid disease progression, the patient was discontinued from the trial on day 63.

Overall, the frequency of grade 3–4 AEs did not increase with increasing dose of TILT-123 (Table 3). In total, 7 serious adverse events (SAEs) were registered, 4 related to TILT-123 and 3 related to TIL treatment.

All registered treatment-related AEs and their frequencies are presented in Table S2.

Efficacy of the treatment

Best overall responses (BORs) evaluated by RECIST (Response Evaluation Criteria in Solid Tumors) 1.1 after TIL infusion (day 78 or later) and PFS are illustrated in Figures 2A and 2B, respectively. All imaging data are presented in Table S3.

The overall response rate (ORR) after TIL infusion was 11.7% (2/17). On day 78, eleven patients had progressive disease (PD) and were not eligible for additional TILT-123 treatment in the extension phase of the trial. The median time to progression was 78 days (range 64–961). On day 78, disease control (stable disease [SD] or partial response [PR]) was observed in six patients (35%, 4 SD, 2 PR), whereof four had non-cutaneous melanoma subtypes (two uveal and two mucosal). Of these, three patients (101.07, 101.14, and 101.15) continued treatment with TILT-123 every third week in the extension phase of the trial and one patient (101.15) obtained a PR lasting for more than 8 months, whereafter the patient progressed with the appearance of one new lesion. However, the patient continued injections with TILT-123 even after progression because of persistent clinical benefit in both injected and non-injected lesions. Unfortunately, after 14 months, the treatment was discontinued due to PD at multiple sites.

One patient (101.07) with mucosal melanoma obtained a durable CR in the extension phase, still with no evidence of disease almost 3 years from inclusion in the trial. This patient had the residual tumor scar inside the nose resected almost a year after treatment initiation, and the pathology report revealed normal tissue highly inflamed by $CD4^+$ T cells but also with increased $CD8^+$ T cell infiltration. No malignant cells were detected in the

Table 3. AEs grade ≥ 3 related to any of the two study drugs at study end

	Grade	Reg.	*n*	% of patients (CI)	Cohort number
Related to TILT-123					
Fever	3	2	1	5.9% (0.3%–31%)	2
Injection site pain	3	1	1	5.9% (0.3%–31%)	2
Related to TILs					
Worsening of adrenal insufficiency	4	1	1	6.3% (0.3%–32%)	5
TIL reaction	3	1	1	6.3% (0.3%–32%)	5
Chills	3	1	1	6.3% (0.3%–32%)	1
Fever	3	1	1	6.3% (0.3%–32%)	3
Increased ASAT	3	1	1	6.3% (0.3%–32%)	5

Reg, number of registrations; *n*, number of patients; CI, 95% confidence interval. In total, five grade 3 events and one grade 4 event were reported. TILT-123 sample size was 17; TIL sample size was 16.

resected tumor. Interestingly, the TIL infusion product of this patient consisted predominantly of $CD4^+$ T cells. Further, a biopsy from the region of the neck with prior metastases was performed, and no malignant cells were found, thus constituting a pathologically confirmed CR.[15]

Patient 101.14 continued injections with TILT-123 for three months whereafter he progressed. The patient did not start new oncologic treatment and is still alive more than 12 months after discontinuation from the trial. Duration of treatment response/benefit for all patients is illustrated in Figure 3 along with survival status.

Notably, 5/6 patients benefitting from the treatment are still alive today (median 809, range 288–1,484 days from treatment initiation). Of these, two patients received further treatment lines including i.t. IL-2 injections and TIL therapy (101.03) and reinduction with CTLA-4 inhibitor/PD-1 inhibitor and TIL therapy (101.05).

The median OS of all included patients was 620 days (Figure 4).

Metabolic response measured by positron emission tomography (PET) scans (Figure 5) were evaluated as the summed SUV_{max} (maximum standardized uptake value) values of the (up to) 5 most avid lesions on day 36 and 78. Fifteen patients were evaluable after TIL infusion on day 78, and 7 (47%) of these had disease control (stable metabolic disease [SMD], minor metabolic response [MMR], partial metabolic response [PMR], or complete metabolic response [CMR]), including 4 MMRs or PMRs (27%) (Figure 5A). Overall, PET responses mirrored RECIST responses, both in patients benefitting from the treatment (101.07, 101.10, 101.14, and 101.15) and in patients progressing. However, in some patients who showed progression according to the RECIST 1.1 criteria, the SUV_{max} values remained stable on day 78 (102.03, 101.09, and 101.11).

On day 36, before the administration of TILs, RECIST 1.1 and Immune Response Evaluation Criteria in Solid Tumors (iRECIST) analysis showed disease control (SD) in 6/17 (35%) patients, while disease control using PET criteria (SMD, MMR, or PMR) was seen in 10/16 (63%) patients. Interestingly, some of the patients who benefitted from the treatment showed early indication of (pre-TIL-infusion) efficacy on the day 36 scan. This includes patients 101.07, 101.14, and 101.15 for whom a PMR or MMR was obtained on day 36 (Figure 5B), despite SD (101.07 and 101.14) or PD (101.15) according to RECIST 1.1. In patient 15, the metabolic improvement observed on day 36 was not accompanied by a clinical improvement, as the patient experienced increasing pain from several subcutaneous metastases. Interestingly, an objective response and relief of symptoms were observed shortly after TIL infusion in this patient (Figure S2).

In patients responding to therapy (101.07 and 101.15), we observed a reduction in tumor size of both injected and non-injected lesions. In patient 101.07, this was present on day 36 suggesting an abscopal effect of the virus therapy alone.

No differences were observed between dose cohorts when comparing responses and survival.

TIL product characteristics, phenotyping, and antitumor reactivity

The median cell number in the TIL infusion products at the Danish site ($n = 13$) was 48 × 10^9 cells (17.9–86.8). At the French site ($n = 3$), the median cell number was 3 × 10^9 cells (2.09–4.93). The difference in cell numbers can be attributed to the distinct expansion methods used at the Danish and French sites (as detailed in STAR Methods). One of the three French patients received only one of the two planned TIL infusions. Individual cell numbers are presented in Table S1. Phenotypic characterization of the TILs was performed on the 13 Danish patients. Infused products consisted almost exclusively of $CD3^+$ cells (median 99% [94.0–99.9]), while the distribution of $CD8^+$ and $CD4^+$ T cells was highly variable among patients with a median of 17% (range 0.3%–98.3%) $CD8^+$ cells and 46% (1.3%–99.5%) $CD4^+$ cells (Figure S3A). Phenotypic characterization of infusion products (Figure S3B) indicates that most of the infused TILs were effector memory cells (CD45RA− CCR7−), while only a smaller proportion were terminally differentiated effector cells (Effector Memory CD45RA+ [EMRA]) (CD45RA+, CCR7−). Overall, TIL product exhibited high expression of CD39 in both $CD4^+$ and $CD8^+$ T cell subsets and high expression of CD28 particularly within the $CD4^+$ T cell population, while expression of PD-1 was nearly undetectable in both $CD4^+$ and $CD8^+$ TILs (Figure S3B)

Multicytokine intracellular staining showed $CD8^+$ reactive T cells toward autologous tumor digest, tumor cell line, and tumor cell line pretreated with interferon (IFN)g in patients 101.04, 101.09, 101.11, and 101.15 (Figure S4). Interestingly, the infusion product of two of these patients (101.09 and 101.11) consisted almost exclusively of $CD4^+$ T cells. Also of interest is patient 101.02, where a high number of tumor digest-reactive $CD4^+$ T cells were observed.

Treatment-related changes in populations of PBMCs

Peripheral blood mononuclear cells (PBMCs) collected on days −14, 8, 36, and 64 of the treatment schedule were phenotypically characterized via flow cytometry (flow cytometry panels are shown in Table S4). Nearly all immune populations were relatively stable during the treatment course, and no specific alterations were seen in relation to virus injections or TIL therapy.

CellPress
OPEN ACCESS

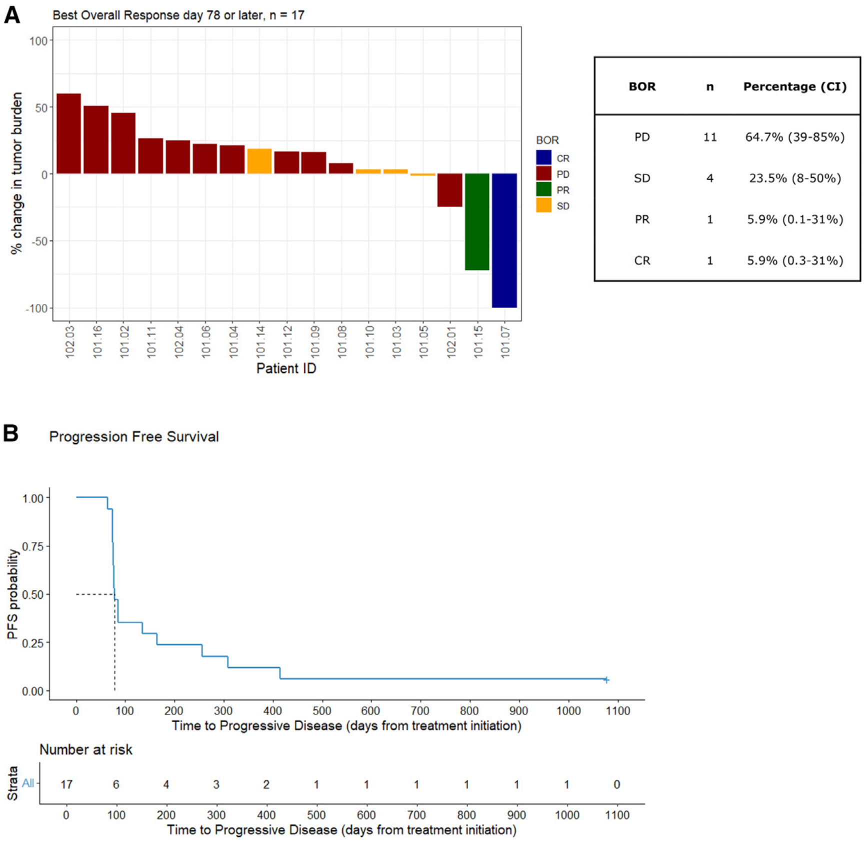

BOR	n	Percentage (CI)
PD	11	64.7% (39-85%)
SD	4	23.5% (8-50%)
PR	1	5.9% (0.1-31%)
CR	1	5.9% (0.3-31%)

Figure 2. Efficacy of the treatment

(A) Best overall response (BOR). Percentage change in tumor burden was evaluated by RECIST 1.1. CR, complete response; PR, partial response; SD, stable disease; PD, progressive disease. CI, 95% confidence interval.

(B) Progression-free survival, n = 17. Median PFS (mPFS) = 78 days.

However, PBMCs collected at day 8 of the treatment interestingly exhibited a significantly higher expression of PD-1, compared to all other time points (Figure S5A).

Ex vivo IFNg Enzyme-Linked ImmunoSpot (ELISpot) testing the reactivity of PBMCs to autologous tumor showed a tendency of increased reactivity in some patients (101.03, 101.04, 101.06, 101.12, and 101.15) after TIL infusion (Figure S5B), but no significant changes were detected. This tendency was not seen when autologous tumor cells were pre-stimulated with IFNg. In two patients (101.03 and 101.09), the reactivity of PBMCs toward autologous tumor cell line was high at baseline but did not alter significantly during the trial (Figure S5B).

Cytokine analyses on peripheral blood revealed a systemic effect of both TILT-123 and TILs in some patients. An exemplary serum cytokine measurement for patient 101.03 is shown in Figure S6, where increases in granzyme B and TNF are observed after i.v. TILT-123 administration as well as TIL administration.

Neutralizing antibodies

Neutralizing antibodies (NAbs) targeting the adenoviral vector have been considered a detrimental factor for OV therapy, due to neutralization of virions, which might limit systemic spreading. We assessed antibody responses against TILT-123 with a functional NAb assay. At baseline, 6/14 patients had no measurable NAbs against TILT-123, while 8 patients had a measurable titer (Figure S7A). NAb titers increased in all patients across the trial. Baseline NAb titers did not predict disease control (Figure S7B, n = 16 patients), OS, time or PFS. A higher end-of-trial NAb titer was associated with a better computed tomography (CT) response on day 36 (Figure S7C, n = 15 patients).

Virus presence in blood, urine, and saliva

The presence of TILT-123 was assayed from blood, urine, and saliva with quantitative polymerase chain reaction (qPCR). Regarding blood, TILT-123 was detected in the blood 1 and 16 h after intravenous and intratumoral dosing (Figures S8A and S8B, n = 10 patients), suggestive of systemic presence of TILT-123 not only after intravenous but also after intratumoral dosing.

Regarding urine and saliva, occasional qPCR positivity was seen in selected patients (101.02, 101.07, 101.09, 102.01, and 102.03). Saliva qPCR positivity was likely explained by tumor location proximity to salivary glands or oral cavity.

DISCUSSION

The prognosis of patients suffering from MM has improved dramatically with the introduction of ICIs and targeted therapies. However, in patients showing primary resistance to ICIs, survival rates are still very low.[16] Moreover, in rarer melanoma subtypes, such as mucosal and uveal melanoma, treatment options are even more limited, and the prognosis remains poor.[17] With the recent FDA approval of TIL therapy for cutaneous melanoma patients,[1] there is a possibility to improve survival in patients not benefitting from ICIs. Despite this promising step forward, the complexity and safety profile of traditional TIL therapy make it clear that new treatment modalities should focus not only on efficacy but also on ways to reduce toxicity and improve accessibility.

TILT-T215 confirms the safety results from the monotherapy trial TUNIMO[14] that TILT-123 is safe and feasible to administer. The combination with TIL therapy did not increase the toxicity of TILT-123 itself, and AEs related to the TIL infusion were generally low grade with only few grade ≥3 events. Thus, compared to classical TIL therapy, combinational therapy of TILT-123 with TILs has an attractive toxicity profile, which could potentially broaden the treatments' accessibility. The trial further demonstrates that injection with TILT-123 is feasible at multiple tumor sites with few injection-related side effects. Finally, the

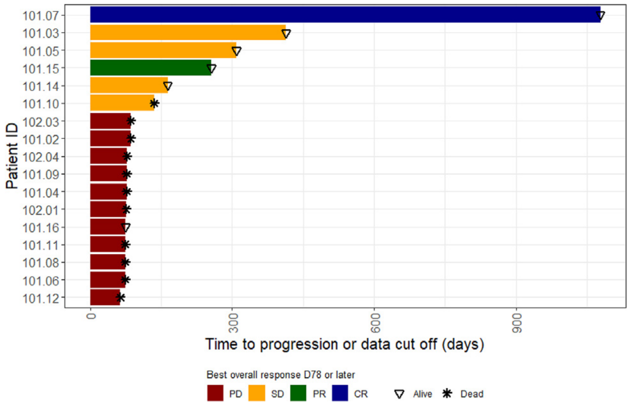

Figure 3. Swimmer plot
Swimmer plot illustrating time to progressing or data cutoff for each patient separately. Colors indicate best overall response on day 78 or later. Stars indicate patients who have died. Triangles indicate patients still alive.

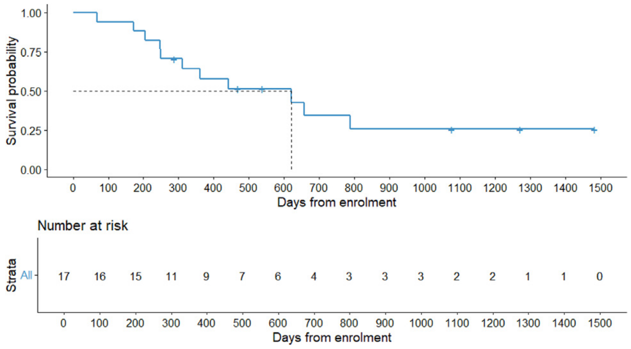

Figure 4. Overall survival
Median overall survival (mOS) = 620 days.

frequency or severity of TILT-123-related AEs did not increase with increasing TILT-123 dose, and the maximum tolerated dose (MTD) was not reached.

Overall, TILT-123 treatment, alone or combined with TILs, is considered a safe and feasible treatment modality in patients with MM.

TILT-T215 rethinks the traditional TIL therapy setup by replacing the lymphodepleting chemotherapy and high-dose IL-2 with oncolytic adenovirus TILT-123. TILT-123 treatment can be initiated while TILs products are manufactured, which reduces the off-treatment time for the patient. Further, the simplified TIL schedule makes it possible to administer TILs in an outpatient setting, which is favored by most patients and represents a cost reduction compared to traditional TIL therapy.

With an ORR of 11.7% and a disease control rate of 35%, TILT-T215 demonstrates that responses to TIL therapy in combination with OV can be obtained without lymphodepletion and IL-2. Of the six patients benefitting from the treatment, three had non-cutaneous melanoma subtypes (two uveal and one mucosal). Due to the combinatorial approach, it is challenging to distinguish the individual contributions of TILT-123 and TILs to responses. In patient 07, tumor shrinkage started *before* the administration of TILs, suggesting clinical efficacy of TILT-123 monotherapy.[15] More extensive investigation of this case suggested that the response was driven by increased tumor infiltration of $CD4^+$ and $CD8^+$ cells and that this process might have been induced by TILT-123 and accelerated by the infusion of TILs.[15] Importantly, this patient showed evolving response to the treatment in both injected and non-injected tumors on the CT scan prior to TIL infusion. This is in line with earlier reports that TILT-123 can induce abscopal effects.[18] Moreover, one patient with uveal melanoma had long-lasting SD (>10 months) despite being off treatment from day 78. Interestingly, in this patient, TIL expansion failed and TILT-123 was administered as monotherapy. Again, this supports the notion that the virus itself can result in antitumor effects. Future analyses of changes in the TME are required to understand the mechanisms behind the local effect and spreading of the virus.

In patient 15, tumor shrinkage was not observed until shortly after TIL infusion as shown in Figure S2. This, along with the high tumor reactivity of the TILs from patient 15, indicates that the TIL product was necessary for the response to develop. Given that TIL therapy without pre- and postconditioning regimens has rarely been associated with long-lasting responses, it can be speculated that TILT-123 supported TIL engraftment and functionality in this patient. However, translational data to support this hypothesis are warranted.

Further, the cytokine measurement from patient 101.03 demonstrates that both TILT-123 and TILs exerted systemic effects, as indicated by increased levels of Granzyme B and TNF in peripheral blood.

The efficacy of OVs is debated, as no studies have so far demonstrated convincing efficacy of OV monotherapy. Recently, updated data from the IGNYTE (ClinicalTrials.gov identifier: NCT03767348) trial showed an ORR of 31.4% in PD-1-resistant MM patients treated with an oncolytic HSV in combination with a PD-1 inhibitor.[19] Tumor shrinkage was observed in injected as well as non-injected lesions. Given that these patients had previously progressed on PD-1 inhibitor therapy, their chance of responding to PD-1 inhibitor re-treatment must be considered low. However, the contribution of the PD-1 inhibition to the observed responses is unclear, and it is possible that the combination with OV was sufficient to overcome the PD-1 resistance. This finding supports the notion that the greatest potential for OV therapy lies in combination with other treatments. Preclinical data showing that TILT-123 enhances TIL efficacy[9] support the combinatorial approach used in TILT-T215. Furthermore, a recent clinical trial treating solid cancers with TILT-123 monotherapy did not result in long-lasting objective responses in most patients.[14] Overall, the specific contributions of each treatment component in TILT-T215 remain unknown, necessitating further research to dissect the roles of TILT-123 and TILs.

The patient population in TILT-T215 differs from other T cell trials in several ways. Importantly, the trial included melanomas of any histology, and hard-to-treat melanoma subtypes (uveal and mucosal) constituted 53% (9/17) of the cohort. Further, patients included were heavily pretreated, and only 35% of the patients had received just one line of therapy for metastatic disease.

In a previous phase 3 trial, TIL therapy was tested against ipilimumab in patients with metastatic cutaneous melanoma.

CellPress
OPEN ACCESS

A

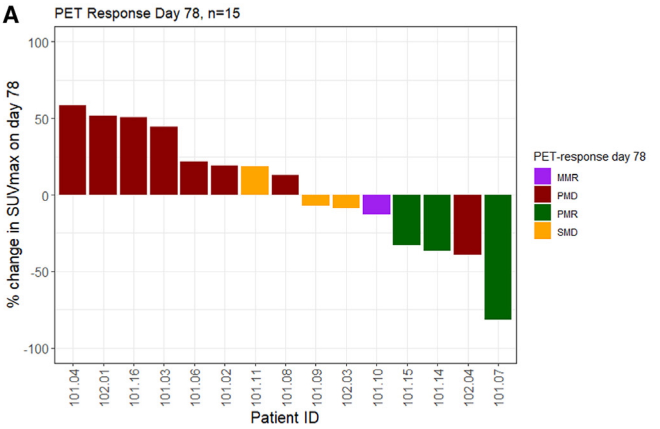

PET response D78	n	Percentage (CI)
PMD	8	53.3% (27-78%)
SMD	3	20% (5-49%)
MMR	1	6.7% (0.3-34%)
PMR	3	20% (5-49%)

B

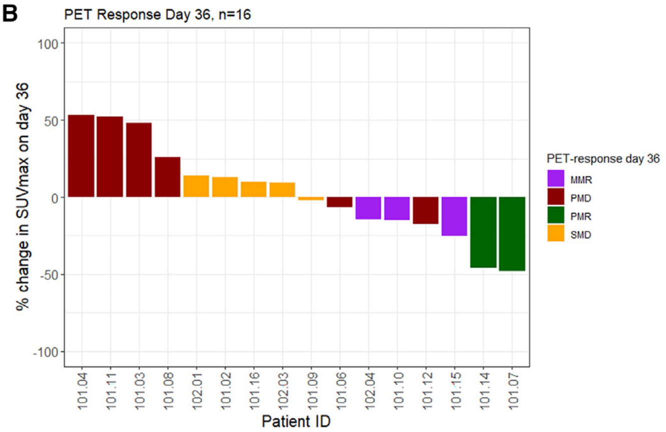

PET response D36	n	Percentage (CI)
PMD	6	37.5% (16-64%)
SMD	5	31.3% (12-59%)
MMR	3	18.8% (5-46%)
PMR	2	12.5% (2-40%)

Figure 5. PET response day 36 and 78

PMR, partial metabolic response; MMR, minor metabolic response; SMD, stable metabolic disease; PMD, progressive metabolic disease; CI, 95% confidence interval.

(A) Percentage change in SUVmax evaluated on day 78.

(B) Percentage change in SUVmax evaluated on day 36.

TIL therapy showed superiority with a response rate of 49%, including 20% with CR.[20] In this phase 3 trial, TILs were administered as 2nd line treatment after progression on 1st line PD-1 inhibitor. The efficacy of TIL therapy in heavily pretreated patients is expected to be lower, and it is well known that the number of prior treatment lines, tumor burden, and high levels of lactate dehydrogenase (LDH) influence response to TIL therapy negatively.[21]

The recent FDA approval of lifileucel[1] was based on results from a phase 2 trial, including patients progressing after more than one line of treatment (median of 3 lines), and an ORR of 31.4% was reported.[22,23] Moreover, encouraging results with an ORR of 50% were recently reported in a subgroup of these patients with metastatic mucosal melanoma (G.-U. Grigoleit, 2023, ESMO Congress 2023, abstract). The trial did not include uveal melanomas; thus, the trial population is not completely comparable with the population in TILT-T215. Still, the response rates exceed the results presented here, suggesting that the treatment schedule of TILT-123 used in TILT-T215 cannot fully replace the conventional conditioning regimens of "classic" TIL therapy.

In the two patients with objective response (101.07 and 101.15), we observed an early indication of benefit in the SUV_{max} values (PMR or MMR) on day 36. Preclinical and clinical data on OV treatment suggest that PET scans might be a better evaluation tool compared to RECIST 1.1 for this therapy.[24] Indeed, extending this notion to other immunotherapies, it was reported that an early PET response on immunotherapy with ICI might be a predictor of treatment efficacy.[25] Interestingly, this phenomenon has also recently been described in relation to TIL therapy (T.H. Borch et al., 2023, ESMO Immuno-oncology Congress 2023, abstract). However, in the current study with a limited number of patients, it cannot be conclusively demonstrated that early PET response predicts later RECIST response. It is nevertheless likely that fluorodeoxyglucose (FDG) uptake might be a better tool to capture immune-mediated responses, but precise guidelines on how to perform these evaluations are still needed.[25] Also, since activated T cells use a lot of glucose,[24] FDG might not be the optimal tracer.

In the development of OV therapy, it is highly important to identify local or systemic factors that could limit the function of

the virus. Until recently, the presence or development of NAbs toward OVs has been considered an obstacle, presumably hindering immune activation.[26] This view is, however, challenged as it was recently suggested that NAbs are positively associated with OS and PFS in glioblastoma.[27] We found no correlation of NAbs with OS or PFS in this trial. However, in line with the glioblastoma data, higher end-of-trial NAb titer associated positively with better CT response on day 36 (Figure S7C). Clearly, the role of NAbs in OV therapy still needs further exploration.

In the early studies by Rosenberg et al., it became evident that depletion by high-dose chemotherapy of $CD4^+$ $CD25^+$ T-regulatory lymphocytes (Tregs) and cellular sinks of IL-7 and IL-15 is important to obtain durable responses following TIL therapy.[28] Further, the addition of systemic high-dose IL-2 after TIL infusion improves efficacy(34) by stimulating effector-type lymphocytes.[29] None of the agents have shown the same efficacy when administered as monotherapy or in dual combinations,[30] and the three-step treatment is the standard approach in TIL therapy. However, the literature on TIL therapy without lymphodepletion and/or IL-2 is sparse.

TILT-123 intended to mimic the effect of lymphodepleting chemotherapy and high-dose IL-2 by the viral expression of TNF and IL-2 locally in the tumor. The role of TNF in cancer is controversial, but it is well described that at high concentrations it induces tumor regression and recruitment of immune cells through TME remodeling.[31] The specific effect of TNF on $CD4^+$ $CD25^+$ Tregs is debated, and contrasting results have been published,[32–34] indicating that TNF has the potential to act both as an immune suppressor and immune stimulator. Similarly, IL-2 can exert different effects on the immune system depending on the dose, but the high-dose regimen is known to be the most effective in inducing cancer regression.[29] Despite the viral production of TNF and IL-2, immunosuppressive mechanisms in the TME might still limit the potential of TILT-123 combined with TIL therapy. These mechanisms could explain the lack of clinical efficacy in patients where reactivity of $CD8^+$ T cells in the TIL product toward autologous tumor digest, tumor cell line, and tumor cell line pretreated with IFNg was detected. In patients 101.09 and 101.11 with a very limited number of $CD8^+$ T cells in the infusion products, it is also possible that the reactive cells were simply too few to overcome the tumor burden and/or TME-induced immune suppression. Again, analysis on tumor tissue (injected and non-injected) could provide knowledge on virus-induced changes and the viral production of TNF and IL-2.

To more fully overcome the immunosuppression by the TME, a promising next step could be the addition of lymphodepleting chemotherapy prior to TIL infusion. This approach would lead to efficient removal of Tregs and other immune cells competing for important cytokines, but it would also add toxicity to the treatment. We hypothesize that the gain on efficacy would exceed the toxicity cost, especially because the most burdensome part of traditional TIL therapy is the high-dose IL-2 treatment, which should not be needed with IL-2 produced by TILT-123 at tumor site.

Finally, another point for consideration is the timing of the TIL treatment. Due to virus-induced changes in the TME, it is possible that tumor resection *after* the first injections with TILT-123 could lead to a higher frequency of tumor-reactive TILs in the infusion product. This hypothesis could be easily explored in a preclinical setting.

Conclusion

TILT-T215 demonstrates that treatment with TILT-123 is a suitable companion for TILs with the ability to induce objective responses, even in patients with hard-to-treat melanoma subtypes. The treatment is moreover easy to administer, and the treatment-related toxicity is low.

An era of combination immunotherapy beyond ICIs is evolving. TILT-123 is a promising treatment modality with the potential to reduce toxicity and increase the availability of TIL therapy. Further modification of the treatment schedule is pursued to improve the clinical efficacy of the treatment.

Limitations of the study

The trial is limited by the relatively low number of participants, which were carefully selected to fulfill the criteria for inclusion. The study was not powered to detect treatment efficacy, and included patients might not have been representative of the larger MM population. Further, due to the small sample size, the influence of gender on the results cannot be assessed. Thus, efficacy results should be interpreted with caution. As the trial was designed to assess the safety of the combined treatment, TILT-123 and TILs were administered concomitantly. The contribution of each of the administered drugs to responses could not be fully elucidated. This issue needs to be addressed in future trials.

RESOURCE AVAILABILITY

Lead contact

Further information and requests for resources and reagents should be directed to and will be fulfilled by the lead contact Akseli Hemminki, E-mail: akseli.hemminki@helsinki.fi.

Materials availability

This study did not generate new unique reagents.

Data and code availability

- **Statement about the data**: Data reported in this paper will be shared by the lead contact upon request.
- **Statement about the code:** The paper does not report original code.
- **General statement:** Any additional information required to reanalyze the data reported in this paper is available from the lead contact upon request.

ACKNOWLEDGMENTS

We thank Minna Oksanen, Susanna Grönberg-Vähä-Koskela, and Sini Raatikainen for expert assistance. Open access was funded by Helsinki University Library. This study was supported by Jane and Aatos Erkko Foundation, HUCH Research Funds (VTR), Cancer Foundation Finland, Sigrid Jusélius Foundation, Finnish Red Cross Blood Service, TILT Biotherapeutics Oy, and EU Horizon grants 811693 (UNLEASHAD) and 190121193 (I-CREATE). We thank Albert Ehrnrooth and Karl Fazer for research support.

AUTHOR CONTRIBUTIONS

T.J.M. and S.A.P.: preparation, creation, and presentation of the published work, specifically writing the original draft; management of data; application of statistical analysis; and conducting a research and investigation process,

specifically performing the experiments, or data/evidence collection. B.A., E.E., M.D., R.L.E., T.H.B., T.V.K., T.L., H.W.H., C.V., C.L., R.B.H., V.A., A.K., C.K., J.M.S., J.H.A.C., L.H., M.C.W.W., Ö.M., D.C.A.Q., E.J., R.H., S.S., B.D., and V.C.-C.: conducting a research and investigation process, specifically performing the experiments or data/evidence collection. A.H.: acquisition of the financial support for the project leading to this publication; oversight and leadership responsibility for the research activity planning and execution; and preparation and creation of the published work, specifically critical review, commentary, or revision. I.M.S.: management and coordination responsibility for the research activity planning and execution; oversight and leadership responsibility for the research activity planning and execution; and preparation and creation of the published work, specifically critical review, commentary, or revision.

DECLARATION OF INTERESTS

T.J.M. has been a co-investigator in a trial from TILT Biotherapeutics. E.E. has received personal payment or honoraria for lectures, presentations, speakers bureaus, manuscript writing, or educational events from Novartis, Merck, Bristol Myers Squibb, and Pierre Fabre. She has received support for attending meetings and/or travel from Pierre Fabre and Merck. M.D. is an advisor of Achilles Therapeutics and expert group member of Guidepoint LLC and AlphaSights. R.L.E. received institutional drug funding from BMS to investigator-initiated trial. T.H.B. has received personal honoraria for lectures from Bristol Myers Squibb. C.K. is an employee and shareholder at TILT Biotherapeutics. J.M.S. is an employee and stocks/share holder at TILT Biotherapeutics. He received a research grant from TILT Biotherapeutics. He is a co-inventor of patent applications for TILT Biotherapeutics. He received personal payment for consultancy for TILT Biotherapeutics. J.H.A.C. is an employee and shareholder at TILT Biotherapeutics. L.H. is an employee and option holder at TILT Biotherapeutics. D.C.A.Q. is an employee and shareholder at TILT Biotherapeutics. R.H. is an employee at TILT Biotherapeutics. S.S. is an employee and option holder at TILT Biotherapeutics. V.C.-C. is an employee and shareholder at TILT Biotherapeutics. A.H. is a shareholder in Circio Holding ASA and an employee and shareholder at TILT Biotherapeutics Oy. I.M.S. has received personal payment or honoraria for lectures, presentations, speakers bureaus, manuscript writing, advisory board, or educational events from Bristol Myers Squibb, Instil Bio, Mendus, MSD, Novartis, Sanofi, and Takeda. She has stocks/shares in IO Biotech. She has received research grants from Adaptimmune, Asgard Therapeutics, Enara Bio, IO Biotech, Lytix Biopharma, and TILT Biotherapeutics. She has been principal investigator in trials from BMS, Immunocore, Lytix Biopharma, MSD, Novartis, Roche, and TILT Biotherapeutics. The TILT technology is patented by the company TILT Biotherapeutics.

STAR★METHODS

Detailed methods are provided in the online version of this paper and include the following:

- KEY RESOURCES TABLE
- EXPERIMENTAL MODEL AND STUDY PARTICIPANT DETAILS
- METHOD DETAILS
  - Study design
  - Inclusion criteria and exclusion criteria
- QUANTIFICATION AND STATISTICAL ANALYSIS
- ADDITIONAL RESOURCES

SUPPLEMENTAL INFORMATION

Supplemental information can be found online at https://doi.org/10.1016/j.xcrm.2025.102016.

Received: August 29, 2024
Revised: September 22, 2024
Accepted: February 14, 2025
Published: March 18, 2025

REFERENCES

1. Kwong, M.L.M., and Yang, J.C. (2024). Lifileucel: FDA-approved T-cell therapy for melanoma. The Oncologist *29*, 648–650. https://www.fda.gov/news-events/press-announcement.
2. Rahman, M.M., and McFadden, G. (2021). Oncolytic viruses: Newest frontier for cancer immunotherapy. Cancers *13*, 5452.
3. Guedan, S., and Alemany, R. (2018). CAR-T cells and oncolytic viruses: Joining forces to overcome the solid tumor challenge. Front. Immunol. *9*, 2460.
4. Chesney, J.A., Ribas, A., Long, G.V., Kirkwood, J.M., Dummer, R., Puzanov, I., Hoeller, C., Gajewski, T.F., Gutzmer, R., Rutkowski, P., et al. (2023). Randomized, Double-Blind, Placebo-Controlled, Global Phase III Trial of Talimogene Laherparepvec Combined with Pembrolizumab for Advanced Melanoma. J. Clin. Oncol. *41*, 528–540.
5. Wongariyapak, A., Roulstone, V., Melcher, A.A., Pedersen, M., and Harrington, K.J. (2023). Combination strategies incorporating oncolytic viruses and immune checkpoint inhibitors for advanced melanoma: what is the evidence? Ann. Transl. Med. *11*, 369.
6. Stahlie, E.H.A., Mulder, E.E.A.P., Reijers, S., Balduzzi, S., Zuur, C.L., Klop, W.M.C., van der Hiel, B., Van de Wiel, B.A., Wouters, M.W.J.M., Schrage, Y.M., et al. (2022). Single agent Talimogene Laherparepvec for stage IIIB-IVM1c melanoma patients: A systematic review and meta-analysis. Crit. Rev. Oncol. Hematol. *175*, 103705.
7. Nguyen, H.M., Bommareddy, P.K., Silk, A.W., and Saha, D. (2022). Optimal timing of PD-1 blockade in combination with oncolytic virus therapy. Semin. Cancer Biol. *86*, 971–980.
8. Cervera-Carrascon, V., Quixabeira, D.C.A., Havunen, R., Santos, J.M., Kutvonen, E., Clubb, J.H.A., Siurala, M., Heiniö, C., Zafar, S., Koivula, T., et al. (2020). Comparison of Clinically Relevant Oncolytic Virus Platforms for Enhancing T Cell Therapy of Solid Tumors. Mol. Ther. Oncolytics *17*, 47–60.
9. Havunen, R., Siurala, M., Sorsa, S., Grönberg-Vähä-Koskela, S., Behr, M., Tähtinen, S., Santos, J.M., Karell, P., Rusanen, J., Nettelbeck, D.M., et al. (2017). Oncolytic Adenoviruses Armed with Tumor Necrosis Factor Alpha and Interleukin-2 Enable Successful Adoptive Cell Therapy. Mol. Ther. Oncolytics *4*, 77–86.
10. Siurala, M., Havunen, R., Saha, D., Lumen, D., Airaksinen, A.J., Tähtinen, S., Cervera-Carrascon, V., Bramante, S., Parviainen, S., Vähä-Koskela, M., et al. (2016). Adenoviral Delivery of Tumor Necrosis Factor-α and Interleukin-2 Enables Successful Adoptive Cell Therapy of Immunosuppressive Melanoma. Mol. Ther. *24*, 1435–1443.
11. Siurala, M., Vähä-Koskela, M., Havunen, R., Tähtinen, S., Bramante, S., Parviainen, S., Mathis, J.M., Kanerva, A., and Hemminki, A. (2016). Syngeneic syrian hamster tumors feature tumor-infiltrating lymphocytes allowing adoptive cell therapy enhanced by oncolytic adenovirus in a replication permissive setting. OncoImmunology *5*, e1136046.
12. Santos, J.M., Cervera-Carrascon, V., Havunen, R., Zafar, S., Siurala, M., Sorsa, S., Anttila, M., Kanerva, A., and Hemminki, A. (2018). Adenovirus Coding for Interleukin-2 and Tumor Necrosis Factor Alpha Replaces Lymphodepleting Chemotherapy in Adoptive T Cell Therapy. Mol. Ther. *26*, 2243–2254.
13. Santos, J.M., Havunen, R., Siurala, M., Cervera-Carrascon, V., Tähtinen, S., Sorsa, S., Anttila, M., Karell, P., Kanerva, A., and Hemminki, A. (2017). Adenoviral production of interleukin-2 at the tumor site removes the need for systemic postconditioning in adoptive cell therapy. Int. J. Cancer *141*, 1458–1468.
14. Pakola, S.A., Peltola, K.J., Clubb, J.H.A., Jirovec, E., Haybout, L., Kudling, T.V., Alanko, T., Korpisaari, R., Juteau, S., Jaakkola, M., et al. (2024). Safety, efficacy, and biological data of T cell-enabling oncolytic adenovirus TILT-123 in advanced solid cancers from the TUNIMO monotherapy phase I trial. Clin. Cancer Res. *30*, 3715–3725. https://doi.org/10.1158/1078-0432.ccr-23-3874.

15. Monberg, T.J., Kudling, T., Albieri, B., Pakola, S., Ellebaek, E., Donia, M., Eefsen, R., von Buchwald, C., Kistler, C., Santos, J., et al. (2024). Durable complete response after combined treatment with tumor-infiltrating lymphocytes and oncolytic adenovirus (TILT-123) in a patient with metastatic mucosal melanoma. Immuno-Oncology and Technology *24*, 100726. https://doi.org/10.1016/j.iotech.2024.100726.

16. Amaral, T., Seeber, O., Mersi, E., Sanchez, S., Thomas, I., Meiwes, A., Forschner, A., Leiter, U., Eigentler, T., Keim, U., and Garbe, C. (2020). Primary resistance to pd-1-based immunotherapy— a study in 319 patients with stage iv melanoma. Cancers *12*, 1027.

17. Klemen, N.D., Wang, M., Rubinstein, J.C., Olino, K., Clune, J., Ariyan, S., Cha, C., Weiss, S.A., Kluger, H.M., and Sznol, M. (2020). Survival after checkpoint inhibitors for metastatic acral, mucosal and uveal melanoma. J. Immunother. Cancer *8*, e000341.

18. Havunen, R., Santos, J.M., Sorsa, S., Rantapero, T., Lumen, D., Siurala, M., Airaksinen, A.J., Cervera-Carrascon, V., Tähtinen, S., Kanerva, A., and Hemminki, A. (2018). Abscopal Effect in Non-injected Tumors Achieved with Cytokine-Armed Oncolytic Adenovirus. Mol. Ther. Oncolytics *11*, 109–121.

19. Wong M.K., Sacco, J.J., Robert, C., Michels, J., Bowles, T.L., In, G.K., Tsai, K.K., Lebbe, C., Gaudy-Marqueste, C., Muñoz Couselo, E., et al., Efficacy and safety of RP1 combined with nivolumab in patients with anti–PD-1–failed melanoma from the IGNYTE clinical trial. (2024).

20. Rohaan, M.W., Borch, T.H., van den Berg, J.H., Met, Ö., Kessels, R., Geukes Foppen, M.H., Stoltenborg Granhøj, J., Nuijen, B., Nijenhuis, C., Jedema, I., et al. (2022). Tumor-Infiltrating Lymphocyte Therapy or Ipilimumab in Advanced Melanoma. N. Engl. J. Med. *387*, 2113–2125. https://doi.org/10.1056/NEJMoa2210233.

21. Fernández, V.A. (2024). Biomarkers for response to TIL therapy : a comprehensive review. J Immunother Cancer *12*, e008640. https://doi.org/10.1136/jitc-2023-008640.

22. Chesney, J. (2022). Efficacy and safety of lifileucel, a one-time autologous tumor-infiltrating lymphocyte (TIL) cell therapy, in patients with advanced melanoma after progression on immune checkpoint inhibitors and targeted therapies: Pooled analysis of consecutive cohorts. J Immunother Cancer *10*, 1–14.

23. Medina, T. (2023). LONG-TERM EFFICACY AND SAFETY OF LIFILEUCEL TUMOR-INFILTRATING LYMPHOCYTE (TIL) CELL THERAPY IN PATIENTS WITH ADVANCED MELANOMA: A 4-YEAR ANALYSIS OF THE C-144–01 STUDY. J Immunother Cancer *11*, 2023.

24. Koski, A., Ahtinen, H., Liljenback, H., Roivainen, A., Koskela, A., Oksanen, M., Partanen, K., Laasonen, L., Kairemo, K., Joensuu, T., and Hemminki, A. (2013). [18F]-fluorodeoxyglucose positron emission tomography and computed tomography in response evaluation of oncolytic adenovirus treatments of patients with advanced cancer. Hum. Gene Ther. *24*, 1029–1041.

25. Costa, L.B., Queiroz, M.A., Barbosa, F.G., Nunes, R.F., Zaniboni, E.C., Ruiz, M.M., Jardim, D., Gomes Marin, J.F., Cerri, G.G., and Buchpiguel, C.A. (2021). Reassessing patterns of response to immunotherapy with pet: From morphology to metabolism. Radiographics *41*, 120–143.

26. Groeneveldt, C., van den Ende, J., and van Montfoort, N. (2023). Preexisting immunity: Barrier or bridge to effective oncolytic virus therapy? Cytokine Growth Factor Rev. *70*, 1–12.

27. Ling, A.L., Solomon, I.H., Landivar, A.M., Nakashima, H., Woods, J.K., Santos, A., Masud, N., Fell, G., Mo, X., Yilmaz, A.S., et al. (2023). Clinical trial links oncolytic immunoactivation to survival in glioblastoma. Nature *623*, 157–166.

28. Gattinoni, L., Finkelstein, S.E., Klebanoff, C.A., Antony, P.A., Palmer, D.C., Spiess, P.J., Hwang, L.N., Yu, Z., Wrzesinski, C., Heimann, D.M., et al. (2005). Removal of homeostatic cytokine sinks by lymphodepletion enhances the efficacy of adoptively transferred tumor-specific CD8+ T cells. J. Exp. Med. *202*, 907–912.

29. Raeber, M.E., Sahin, D., Karakus, U., and Boyman, O. (2023). A systematic review of interleukin-2-based immunotherapies in clinical trials for cancer and autoimmune diseases. EBioMedicine *90*, 104539.

30. Rosenberg, S.A., Spiess, P., and Lafreniere, R. (1986). A new approach to the adoptive immunotherapy of cancer with tumor-infiltrating lymphocytes. Science *233*, 1318–1321.

31. Wang, X., and Lin, Y. (2008). Tumor necrosis factor and cancer, buddies or foes? Acta Pharmacol. Sin. *29*, 1275–1288.

32. Valencia, X., Stephens, G., Goldbach-Mansky, R., Wilson, M., Shevach, E.M., and Lipsky, P.E. (2006). TNF downmodulates the function of human CD4+ CD25hi T-regulatory cells. Blood *108*, 253–261.

33. Montfort, A., Colacios, C., Levade, T., and Andrieu-abadie, N. (2019). The TNF Paradox in Cancer Progression and Immunotherapy. Front. Immunol. *10*, 1–5.

34. Jung, M.K., Lee, J.S., Kwak, J.E., and Shin, E.C. (2019). Tumor Necrosis Factor and Regulatory T Cells. Yonsei Med. J. *60*, 126–131.

35. Donia, M., Larsen, S.M., Met, Ö., and Svane, I.M. (2014). Simplified protocol for clinical-grade tumor-infiltrating lymphocyte manufacturing with use of the Wave bioreactor. Cytotherapy *16*, 1117–1120.

36. Jotereau, F., Pandolfino, M.C., Boudart, D., Diez, E., Dreno, B., Douillard, J.Y., Muller, J.Y., and LeMevel, B. (1991). High-fold expansion of human cytotoxic T-lymphocytes specific for autologous melanoma cells for use in immunotherapy. J. Immunother. *10*, 405–411.

37. Pandolfino, M.C., Labarrière, N., Tessier, M.H., Cassidanius, A., Bercegeay, S., Lemarre, P., Dehaut, F., Dréno, B., and Jotereau, F. (2001). High-scale expansion of melanoma-reactive TIL by a polyclonal stimulus: Predictability and relation with disease advancement. Cancer Immunol. Immunother. *50*, 134–140.

38. Draghi, A., Presti, M., Jensen, A.W.P., Chamberlain, C.A., Albieri, B., Rasmussen, A.C.K., Andersen, M.H., Crowther, M.D., Svane, I.M., and Donia, M. (2023). Uncoupling CD4+ TIL-Mediated Tumor Killing from JAK-Signaling in Melanoma. Clin. Cancer Res. *29*, 3937–3947.

39. Wickham, H. (2016). Data analysis (Springer).

40. Alboukadel Kassambara, M.K., Biecek, P., and Fabian, S. (2021). SmulTCan: A Shiny application for multivariable survival analysis of TCGA data with gene sets. Computers in Biology and Medicine *137*, 104793. https://doi.org/10.32614/CRAN.package.survminer.

41. Wickham, H., Averick, M., Bryan, J., Chang, W., McGowan, L., François, R., Grolemund, G., Hayes, A., Henry, L., Hester, J., et al. (2019). Welcome to the tidyverse. J. Open Source Softw. *4*, 1686.

42. Eisenhauer, E.A., Therasse, P., Bogaerts, J., Schwartz, L.H., Sargent, D., Ford, R., Dancey, J., Arbuck, S., Gwyther, S., Mooney, M., et al. (2009). New response evaluation criteria in solid tumours: Revised RECIST guideline (version 1.1). Eur. J. Cancer *45*, 228–247.

STAR★METHODS

KEY RESOURCES TABLE

REAGENT or RESOURCE	SOURCE	IDENTIFIER
Antibodies		
BD Horizon™ BV421 Mouse Anti-Human CD107A	BD Biosciences	562623; RRID:AB_2737685
BD GolgiStop™ Protein Transport Inhibitor (Containing Monensin)	BD Biosciences	554724; RRID:AB_2869012
BD GolgiPlug™ Protein Transport Inhibitor (Containing Brefeldin A)	BD Biosciences	555029; RRID:AB_2869014
BD Horizon™ BV711 Mouse Anti-Human CD4	BD Biosciences	563028; RRID:AB_2737961
CD8 Monoclonal Antibody (3B5), Qdot™ 605	Thermo Fisher Scientific, Invitrogen	Q10009; RRID:AB_2556437
BD Pharmingen™ PE-Cy™7 Mouse Anti-Human IFN-γ	BD Biosciences	557643; RRID:AB_396760
BD Pharmingen™ PerCP-Cy™5.5 Mouse Anti-Human HLA-DR	BD Biosciences	560652; RRID:AB_1727529
PE anti-human CD197 (CCR7)	BioLegend - Nordic Biosite	353204; RRID:AB_10913813
BD Horizon™ APC-R700 Mouse Anti-Human CD8	BD Biosciences	565165; RRID:AB_2744457
BD Horizon™ BV421 Mouse Anti-Human CD39	BD Biosciences	563679; RRID:AB_2738368
BD Horizon™ BV510 Mouse Anti-Human CD4	BD Biosciences	562970; RRID:AB_2744424
BD Horizon™ BV605 Mouse Anti-Human CD56	BD Biosciences	562780; RRID:AB_2728700
BD Horizon™ BV650 Mouse Anti-Human CD45RA	BD Biosciences	563963; RRID:AB_2738514
PE/Dazzle™ 594 anti-human CD279 (PD-1)	BioLegend- Nordic Biosite	329940; RRID:AB_2563659
BD Pharmingen™ PE-Cy™5 Mouse Anti-Human CD38	BD Biosciences	555461; RRID:AB_395854
CD127 Antibody, anti-human, REAfinity	Miltenyi Biotach	130-113-417; RRID:AB_2726163
BD Horizon™ BB700 Mouse Anti-Human CD183	BD Biosciences	566532; RRID:AB_2739742
APC anti-human CD194 (CCR4)	BioLegend - Nordic Biosite	359408; RRID:AB_2562429
BD Horizon™ BV711 Mouse Anti-Human CD196 (CCR6)	BD Biosciences	563923; RRID:AB_2738489
BD Horizon™ BV786 Mouse Anti-Human CD3	BD Biosciences	563800; RRID:AB_2744384
BD Horizon™ BV510 Mouse Anti-Human CD4	BD Biosciences	562970; RRID:AB_2744424
BD™ CD25 PE-Cy™7	BD Biosciences	335824; RRID:AB_2868687
BD Horizon™ BV711 Mouse Anti-Human CD19	BD Biosciences	563036; RRID:AB_2737968
APC anti-human CD1c	BioLegend - Nordic Biosite	331524; RRID:AB_10719956
BD Horizon™ BV510 Mouse Anti-Human CD33	BD Biosciences	563257; RRID:AB_2738102
BD Horizon™ BV605 Mouse Anti-Human CD123	BD Biosciences	564197; RRID:AB_2732049
BD Horizon™ BV650 Mouse Anti-Human CD11c	BD Biosciences	563404; RRID:AB_2869490

(Continued on next page)

Continued

REAGENT or RESOURCE	SOURCE	IDENTIFIER
BD Pharmingen™ PE Mouse Anti-Human CD141	BD Biosciences	559781; RRID:AB_397322
PE/XFD700 Anti-human CD16 Antibody	ATT Bioquest	101611P1; https://www.aatbio.com/products/pe-xfd700-anti-human-cd16-antibody-3g8-xfd700-same-structure-to-alexa-fluor-700
BD Horizon™ PE-CF594 Mouse Anti-Human CD14	BD Biosciences	562335; RRID:AB_11153663
Bacterial and virus strains		
TILT-123 (igrelimogene litadenorepvec)	TILT Biotherapeutics	Not applicable
Biological samples		
Tumor Infiltration Lymphocytes	Tumor tissue from included patients. Manufactured by the National Center for Cancer Immune Therapy (CCIT-DK) and Nantes Université, INSERM, CNRS, Immunology and New Concepts in ImmunoTherapy, INCIT, UMR 1302/ EMR6001. F-44000 Nantes, France	Not applicable
Blood	Patients included in the study	Not applicable
Tumor tissue	Patients included in the study	Not applicable
Saliva	Patients included in the study	Not applicable
Software and algorithms		
R software	https://www.r-project.org/	Version 4.3.2
GraphPad Prism	https://www.graphpad.com/features	Version 10.1.2 and Version 9.0.0.121.
NovoExpress software	https://www.agilent.com/en/product/research-flow-cytometry/flow-cytometry-software/novocyte-novoexpress-software-1320805	Version 1.4.1
FlowJo Software	https://www.flowjo.com/solutions/flowjo	Version 10.6.1

EXPERIMENTAL MODEL AND STUDY PARTICIPANT DETAILS

Before enrollment in the trial, all patients provided both written and oral informed consent in accordance with the declaration of Helsinki.

The trial was approved by the Danish National Ethics Committee and the Danish Medicines Agency. All approvals were obtained prior to inclusion of the first patient in the trial.

The trial was monitored by KLIFO, Glostrup, Denmark.

Adults (18–57 years), both females and males, with ICI resistant MM were included in the trial. Criteria for inclusion are provided in METHOD DETAILS. Seventeen patients were included in five dose cohorts and the study followed a standardized 3 + 3 dose escalating model. The study was a single-arm study. Age and gender of included patients is provided in Table 1. Data on participants socioeconomic status and ethnicity was not assessed. The influence of gender on the study results cannot be assessed.

METHOD DETAILS

Study design

TILT-T215 was a phase I, open-label, 3 + 3 dose-escalating multicenter trial for patients with MM (TUNINTIL, NCT04217473). Patients were included in Denmark (n = 14) and in France (n = 3). Included patients received combined treatment with multiple injections with the oncolytic virus TILT-123 (igrelimogene litadenorepvec, Ad5/3-E2F-d24-hTNFa-IRES-hIL2) and treatment with autologous expanded tumor infiltrating lymphocytes (TILs) without preconditioning lymphodepleting chemotherapy or post-conditioning IL-2. Key eligibility criteria were ICI resistant grade III-IV malignant melanoma, an Eastern Cooperative Oncology Group (ECOG) Performance Status of 0 or 1, tumor lesions available for evaluation and intratumoral (I.T.) injections with TILT-123, adequate organ function and no former treatment with TIL therapy.

Included patients received one I.V. dose of TILT-123 followed by five I.T. injections. Tumor resection for TIL therapy was performed before inclusion and TILs were administered on or after day 36, preferably between day 36 and 40.

A Positron Emission Tomography/Computed Tomography (PET/CT) scan was performed on day 36 before TIL-infusion and again on day 78. The final visit was scheduled on day 104. If patients, however, benefitted from the treatment, they could continue treatment with I.T. injections of TILT-123 every third week for up to two years. The treatment schedule is outlined in Figure S1.

Inclusion criteria and exclusion criteria

Inclusion criteria

1. Signed and dated informed consent before any trial-related activities.
2. Male or female, between 1875 years of age (both included).
3. Pathologically confirmed previously treated refractory or recurrent stage 3–4 melanoma, which cannot be treated with curative intent with available therapies.
4. At least one prior line of medical treatment is required (for example checkpoint inhibitors, kinase inhibitors, interleukin-2). Multiple prior therapies (e.g., surgery, checkpoint inhibitors, kinase inhibitors, interleukin-2, interferon, chemotherapy, radiation) are allowed.
5. A > 9 mm tumor (in diameter, typically a minimum of 1 cm3 in volume) without signs of necrosis must be available for biopsy/operation to enable growing of TILs.
6. At least one additional tumor (>14 mm in diameter) must be available for injections and biopsies for correlative analyses. The disease burden must be evaluable. but does not need to fulfil RECIST 1.1.
7. Eligible for adoptive T cell therapy with tumor infiltrating lymphocytes.
8. Adequate hepatic, cardiac and renal functions as following:
   a) Platelets >75 000/mm3.
   b) Hemoglobin ≥100 g/L.
   c) AST and ALT <3 x ULN.
   d) GFR >60 mL/min (Cockcroft-Gault formula).
   e) Leukocytes (WBC) > 3,0.
   f) Bilirubin <1.5 x ULN.
9. Men and women must be willing to use adequate forms of contraception from screening, during the trial, and for a minimum of 90 days after end of treatment, in accordance with the following:
   a. Women of childbearing potential: Barrier contraceptive method (i.e. condom) must be used in addition to one of the following methods: Intrauterine devices or hormonal contraception (oral contraceptive pills, implants, transdermal patches, vaginal rings or long-acting injections).
   b. Women not of childbearing potential: Barrier contraceptive method (i.e. condom) must be used.
   c. Men: Barrier contraceptive method (i.e., condom) must be used.
10. Demonstrated WHO performance score of 0–1 at screening.
11. Life expectancy time longer than 3 months.
12. Capable of understanding and complying with parameters as outlined in the protocol.
13. BRAF negative or positive.

Exclusion criteria

1. Use of immunosuppressive medications (corticosteroids or drugs used in treatment of autoimmune disease). Exempted are the following which can be allowed at screening and during the trial: replacement corticosteroids if e.g., the patient has adrenal insufficiency after prior immunotherapy; pulmonal and topical treatments; up to 20 mg of prednisone/prednisolone.
2. History of another active invasive cancer as judged by the investigator within the past 3 years except basalioma.
3. Treated with any anti-cancer therapy for melanoma 30 days prior to enrolment. Anticancer therapy for melanoma is defined as anti-cancer agents (immunotherapy, signal transduction inhibitors [e.g., BRAF and MEK inhibitors], cytotoxic chemotherapy), radiotherapy and investigational agents. An investigational agent is any drug or therapy that is currently not approved for use in humans.
4. Uncontrolled cardiac or vascular diseases.
5. History of heart attack or cerebral stroke within the previous 12 months before screening or is not recovered from an older heart attack or cerebral stroke.
6. LDH value >3 x ULN.
7. History of hepatic dysfunction, hepatitis or HIV.
8. History of coagulation disorder.
9. Any other medical condition or laboratory abnormality that in the judgment of the principal investigator, may increase the risk associated with study participation or may interfere with interpretation of study results and/or otherwise make the patient inappropriate for entry into this trial.
10. Female patients who are pregnant, breastfeeding or intends to become pregnant.

11. Untreated brain metastases. Treated brain metastases which have not progressed in 3 months prior to screening are allowed.
12. Previously treated with any oncolytic adenovirus that was administered intratumorally.
13. Previously treated with adoptive cell therapy.
14. Allergy to ingredients present in the investigational medicinal products (ingredients are listed in investigator brochures).
15. Administered an investigational medicinal product or device in another clinical trial within 30 days prior to screening.

Administration of TILT-123

Each patient received up to six injections with TILT-123 between day 1 and day 64. The first virus injection was given intravenously, followed by 5 intratumoral injections.

Injected tumors had to be at least 14 mm in diameter and a maximum of 10 lesions could be injected. The total injected volume varied between 1 and 4 mL.

Preparation of the dose was performed bedside by a trained nurse. All personnel handling the virus used protective equipment (coat, gloves, and eye protection). Injections were performed either ultrasound guided by a radiologist or visually guided by a trained physician. TILT-123 is a genetically modified organism (GMO) and waste was handled according to local guidelines and requirements.

Dose escalation

The injections of TILT-123 followed a 3 + 3 dose escalation design as presented below. If a dose-limiting toxicity (DLT) event occurred within the first 3 patients of a cohort the cohort would be expanded with three more patients (6 patients in total). If no further DLT events occur in the additional 3 patients, dose escalation could continue. If the same or similar DLT occurs in 2/6 patients, a maximum tolerated dose (MTD) cohort would be added. The MTD dose would be calculated depending on when in the treatment schedule the DLT occurred.

Cohort	Virus I.V. dose (VP)	Virus I.T. dose (VP)
1 (n = 3)	$3x10^{9}$	$3x10^{9}$
2 (n = 3)	$3x10^{10}$	$3x10^{10}$
3 (n = 3)	$3x10^{11}$	$1x10^{11}$
4 (n = 3)	$1x10^{12}$	$3x10^{11}$
5 (n = 5)	$3x10^{12}$	$3x10^{11}$

Dose escalation. Escalating doses of TILT-123 administered in the 5 cohorts in TILT-T-215. I.V.: Intravenous, I.T.: Intratumoral, VP: Viral Particles.

Generation of tumor infiltrating lymphocytes

Tumor tissue for TIL production was resected during the screening period from day −14 to day 1.

Danish site: At the Danish site resected tumor tissue was placed in culturing media containing RPMI-1640 (Gibco) and 1% Penstrep (Penicillin/Streptomycin, Gibco) and brought immediately to the clean room facility where the tissue was mechanically fragmented into pieces of 1–3 mm^3. Fragments were placed in separate wells with culturing media containing RPMI-1640, 1% Penstrep (penicillin/streptomycin, Gibco), heat-inactivated humanAB serum (Sigma Aldrich), 0.5% Fungizone (BMS) and 6000 IU/mL interleukin-2 (Proleukin, Novartis). Young TILs where then expanded for 3–4 weeks with half of the media replaced with fresh media every second day. After 3–4 weeks young TILs were either frozen or further expanding according to the rapid expansion protocol (REP) as described earlier.[35]

TILs were administered without preconditioning chemotherapy or postconditioning IL-2 on day 37 or later in accordance with the treatment protocol.

French site: At the French site TILs were grown twice from the same tumor tissue. The method has been described previously.[36,37] Briefly, young TILs were grown from tumor fragments for 10–14 days. A fraction of these cells was cryopreserved for later expansion while another fraction underwent more extensive stimulation with irradiated feeder cells and/or IL-2 for 10 + 10 additional days. These cells were administered to the patient on ∼ D37.

The cryopreserved young TILs were expanded similarly after 4 weeks and administered to the patient on ∼ D64.

TILT-123 manufacturing

Construction and production of TILT-123 has been previously described[9]

PET criteria

PET responses were evaluated using the summed SUVmax (maximum standardized uptake value) values of the (up to five) hottest lesions at each timepoint. Results were classified according to the following response categories:

Complete metabolic response (CMR); Complete resolution of (Fluorodeoxyglucose) FDG activity within measurable lesions and all reliably assessable lesions to background levels. No new FDG avid lesions in pattern typical of cancer.*. Lymph nodes may remain metabolically active due to immune response (activated lymphocytes take up FDG).

Partial metabolic response (PMR); >30% reduction in FDG activity measured as the summed SUVmax of measurable lesions (up to five lesions, max 2/organ). No new FDG avid lesions in pattern typical of cancer.*.

Minor metabolic response (MMR); 10–29% reduction in FDG summed SUVmax. No new FDG-avid lesions in pattern typical of cancer.*.

Stable metabolic disease (SMD); 0–9% reduction or up to <30% increase in FDG summed SUVmax. No new FDG-avid lesions in pattern typical of cancer.*.

Progressive metabolic disease (PMD); ≥30% increase in FDG summed SUVmax in pattern typical of tumor, or new clearly FDG-avid clinically significant lesions typical of cancer and associated with CT abnormality most consistent with tumor (≥2 cm in diameter) and clearly not because of inflammation of infection or related to treatment response.

* Increase in metabolic activity in lymph nodes should not result in PMD if no progression is detected elsewhere, since it might reflect immunological activation and not progression. - *"clinically significant lesions in pattern typical of cancer" are defined as lesions associated with a CT abnormality most consistent with cancer (≥2 cm in diameter), and clearly not because of inflammation of infection or related to treatment response.

Immune phenotyping

PBMCs isolated from peripheral blood were phenotypically characterized using three distinct flow cytometry panels (Table S4). Briefly, cells where plated in a V bottom 96 well plate, each well was topped up to 200μL with DPBS (Gibco) and the plate was centrifuged. Following centrifugation, and gentle vortexing, cells were stained with live/dead stain. After a 10 min incubation, cells were further stained with the three antibody mixtures and incubated for 20 min. The cells were washed and pellets resuspended in 100μL of DPBS. The samples were run in a NovoCyte Quanteon Flow Cytometer. Data was analyzed using NovoExpress software v.1.4.1 and GraphPad Prism v9.0.0.121.

PBMCs and infusion product tumor reactivity

Anti-tumor PBMC reactivity was assessed via *ex vivo* IFNg Enzyme-Linked ImmunoSpot (ELISpot). Autologous tumor cells were treated with IFNg three days prior ELISpot set-up. PBMCs were thawed and plated in XVIVO (Lonza) medium the day before. Plates were coated with anti-hIFNg coating antibody (Mabtech) and incubated overnight. Upon ELISpot setup, PBMCs were co-cultured on MultiScreenHTS IP Filter ELISpot plate (Sigma-Aldrich) with autologous tumor cells (with or without IFNg pre-treatment). The following day, plates were washed and incubated with biotinylated anti-hIFNg secondary antibody (Mabtech), followed by Streptavidin-AP(Mabtech) incubation and subsequent addition of BCIP/NBT-plus substrate (Mabtech). Plates were scanned and spots counted using ImmunoSpot S6 Analyzer.

Infusion product reactivity toward autologous tumor cells and tumor digest was performed using a previously described method.[38] Samples were analyzed using a NovoCyte Quanteon Flow Cytometer. Data were analyzed using FlowJo Software v10.6.1 and GraphPad Prism v9.0.0.121.

Virus neutralizing antibodies

Neutralizing antibodies against TILT-123 were assessed with a neutralizing antibody assay described previously.[26] In brief, A549 cells were seeded on 96 well plates. 24 h later, serial dilutions of patients' sera were added on top of the cell, followed by addition of Ad5/3-Luc1. Virus infectivity was assayed 24 h later with luciferase assay (Promega). Neutralizing antibody titer was defined as titer able to neutralize 80% signal relative to mock treated well.

Virus shedding in blood, urine and saliva

Viral shedding in blood, urine and saliva was assayed with quantitative polymerase chain reaction (qPCR) primers targeting the TILT-123 TNFa-IRES-IL2 region. VP/mL calculations from qPCR Ct-values was done with standard curve.

Data analysis

Data analysis was performed in RStudio version 4.3.2 using the packages ggplot2,[39] survminer[40] and tidyverse,[41] and GraphPad Prism version 10.1.2 and version 9.0.0.121.

Outcomes

The primary endpoint was safety of TILT-123 by day 36, prior to TIL administration. Secondary endpoints included safety and tolerability of the combined treatment and tumor response evaluation by standard imaging. Further exploratory endpoints were progression free survival, overall survival, immune response against TILT-123, virus persistence after every TILT-123 injections and virus shedding in urine and saliva.

AEs were registered using the Common Terminology Criteria for Adverse events (CTCAE) version 5 (https://ctep.cancer.gov/protocoldevelopment/electronic_applications/ctc.htm#ctc_50).

Tumor response was evaluated using Response Evaluation Criteria in Solid Tumors (RECIST) version 1.1 and Immune Response Evaluation Criteria in Solid Tumors (iRECIST).[42] Because inflammatory pseudoprogression affects tumor size, which might lead to premature treatment interruption, also PET response was evaluated. A set of practical criteria modified from PERCIST (PET Response Criteria In Solid Tumors) were used.[24] These criteria, as used in this protocol, are outlined in STAR Methods.

The cutoff for all data reported here was 19.08.2024.

QUANTIFICATION AND STATISTICAL ANALYSIS

Survival curves were calculated using the Kaplan-Meier method using RStudio version 4.3.2, package “survminer[40]”, function “ggsurvplot” (Figures 2 and 4) Number of patients (n) = 17. The median progression-free survival (Figure 2) and overall survival (Figure 4) are reported in the figure legend.

95% confidence intervals for the frequency of AEs and for response rates were calculated using proportion testing using RStudio version 4.3.2 Number of patients (n) = 17. Confidence intervals are reported in Tables 2 and 3.

Waterfall plots (Figures 2A and 5) and swimmer plot (Figure 3) were created using RStudio version 4.3.2, using packages “ggplot2[39]” and “tidyverse[41]”. The number of patients (n) is reported in the figures.

Phenotypic characterization of the TIL infusion products from 12 Danish patients (Figure S3B) was created using GraphPad Prism version 9.0.0.121. Data are presented as mean ± standard deviation. Mean is defined as the average value of the data points. Standard deviation is defined as the amount of variation of the data points around the mean.

Changes in the expression of specific markers on PBMCs were evaluated using a two-way ANOVA followed by Tukey’s multiple comparison test using GraphPad Prism version 9.0.0.121(Figure S5A). Number of patients (n) = 13. Results are presented as mean ± standard error of the mean (SEM). Mean is defined as the average value of the data points. SEM is an estimate of how different the sample mean is likely to be from the population mean. The chosen level of significance (p-value) is reported in the figure legend.

The levels of neutralizing antibody titer in patients with and without change in tumor burden were compared using the Mann-Whitney U-test using GraphPad Prism version 10.1.2 (Figures S7B and S7C). The chosen level of significance (p-value) is reported in the figure legend.

Viral particle concentration in peripheral blood for each dose cohorts, at different timepoints post virus injections was compared using two-way ANOVA followed by Fisher’s Least Significant Difference (LSD) test using GraphPad Prism version 10.1.2 (Figure S8B). Total number of patients (n) = 10. Number of patients (n) per dose cohort = 3, except for I.V. dose 1x10ˆ12/I.T. dose 3x10ˆ11 where $n = 1$.

ADDITIONAL RESOURCES

The trial is registered on clinicaltrials.gov: NCT04217473.

Cell Reports Medicine, Volume *6*

Supplemental information

Safety and efficacy of combined treatment with tumor-infiltrating lymphocytes and oncolytic adenovirus TILT-123 in metastatic melanoma

Tine J. Monberg, Santeri A. Pakola, Benedetta Albieri, Eva Ellebaek, Marco Donia, Rikke L. Eefsen, Troels H. Borch, Tatiana V. Kudling, Torben Lorentzen, Helle W. Hendel, Cecilie Vestergaard, Cathrine Lorentzen, Rikke B. Holmstroem, Victor Arias, Amir Khammari, Claudia Kistler, João M. Santos, James H.A. Clubb, Lyna Haybout, Marie C.W. Westergaard, Özcan Met, Dafne C.A. Quixabeira, Elise Jirovec, Riikka Havunen, Suvi Sorsa, Victor Cervera-Carrascon, Brigitte Dreno, Akseli Hemminki, and Inge Marie Svane

TILT-T215 Supplementary Material

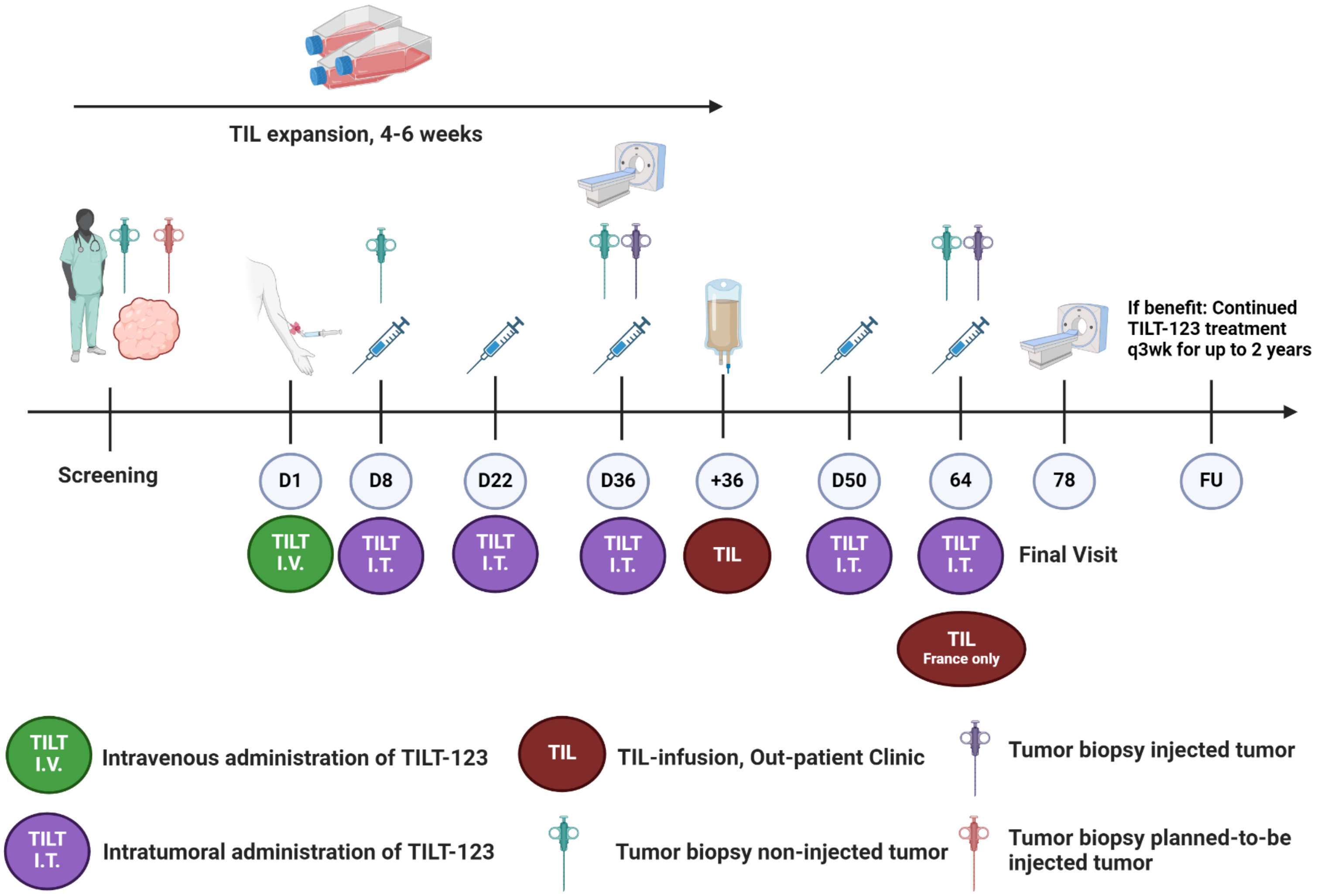

Figure S1: Treatment schedule in TILT-T215. *Tumor tissue for TIL production was resected during the screening and TILs were administered on day 37 or later. Patients received a total of six TILT-123 injections, four before TIL infusion and two post TIL-infusion. If patients benefitted from the treatment, they could continue injections with TILT-123 every third week for up to two years. Related to STAR*METHOD.*

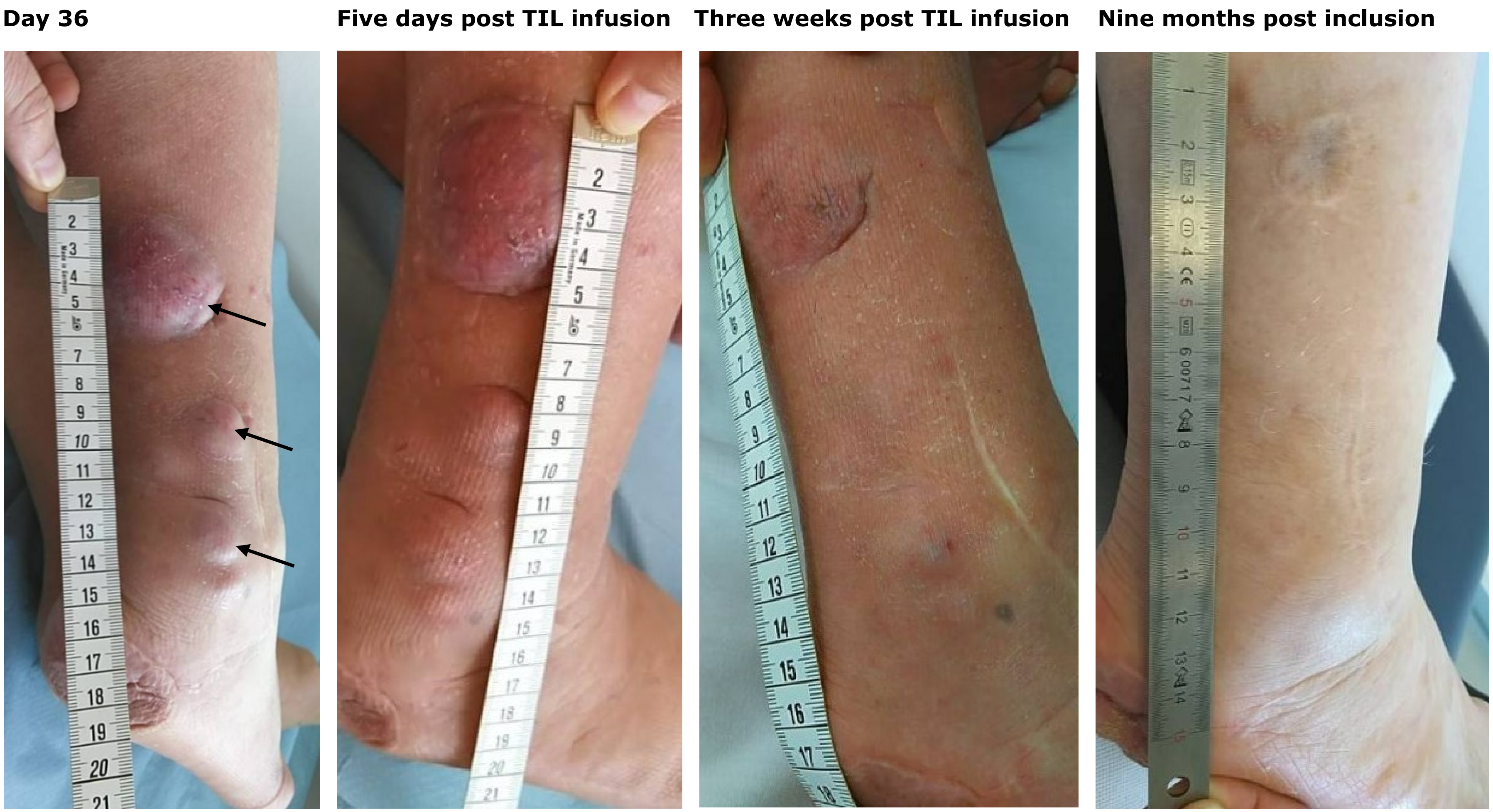

Figure S2: Development of response in patient 101.15. *Patient 101.15 had several subcutaneous metastases on the right lower leg. Before D36 TILT-123 was injected in the three arrow-marked tumors (at least once per tumor). On D36 all subcutaneous metastases had increased in size and the patient experienced increasing pain. Five days after TIL infusion no objective change was observed, but three weeks post TIL infusion all metastases showed a remarkable reduction in size. Nine months post treatment most of these subcutaneous lesions could no longer be visualized. Related to Figure 2.*

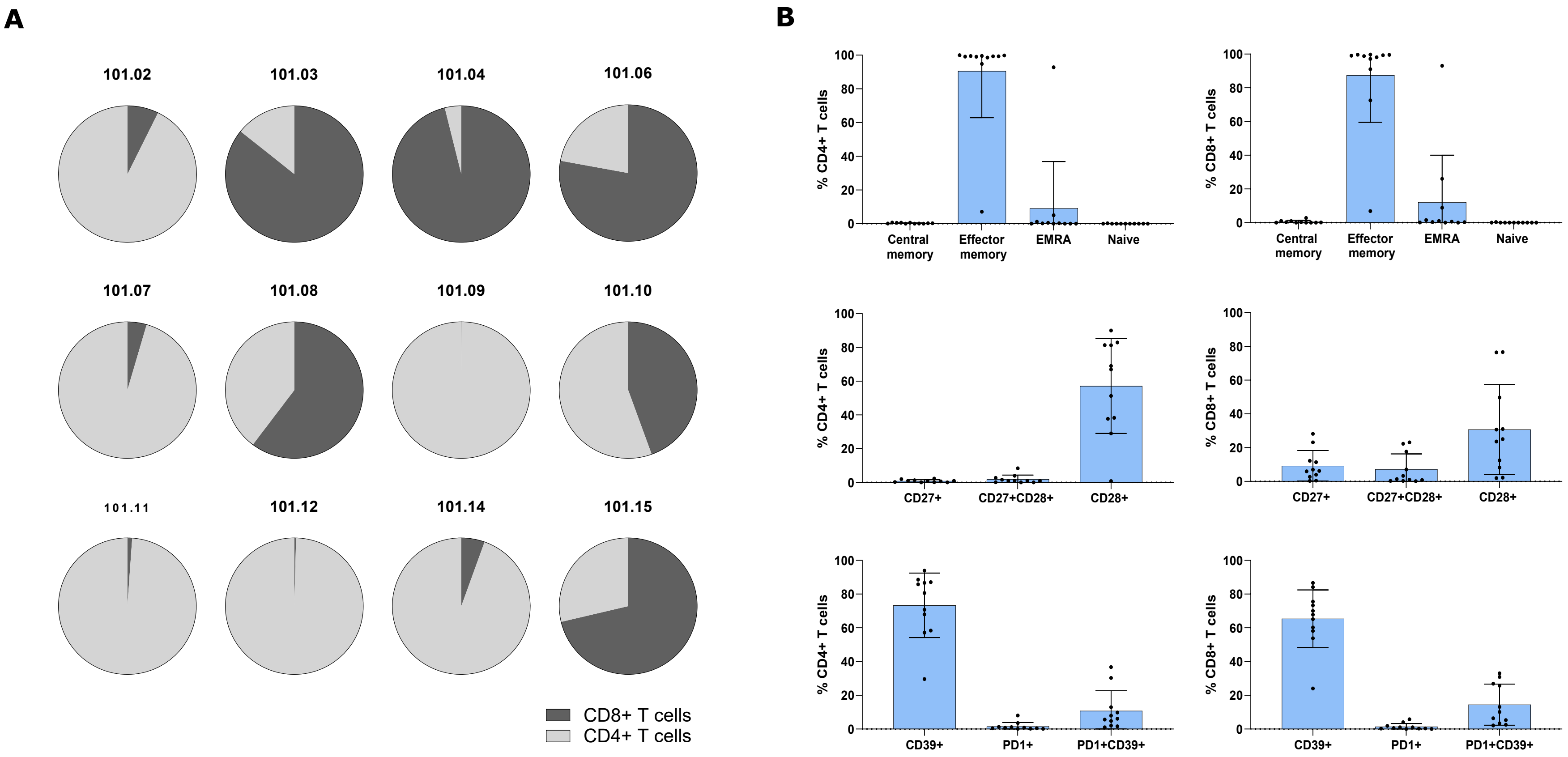

Figure S3: Phenotyping of TIL infusion products. (A) *Distribution of CD4+ and CD8+ T cells out of the living cells in the infusion product from 12 patients treated at the Danish site.* ***(B)*** *Phenotypic characterization of the TIL infusion products from 12 Danish patients. Data are presented as mean ± standard deviation (SD). Related to STAR*METHOD*

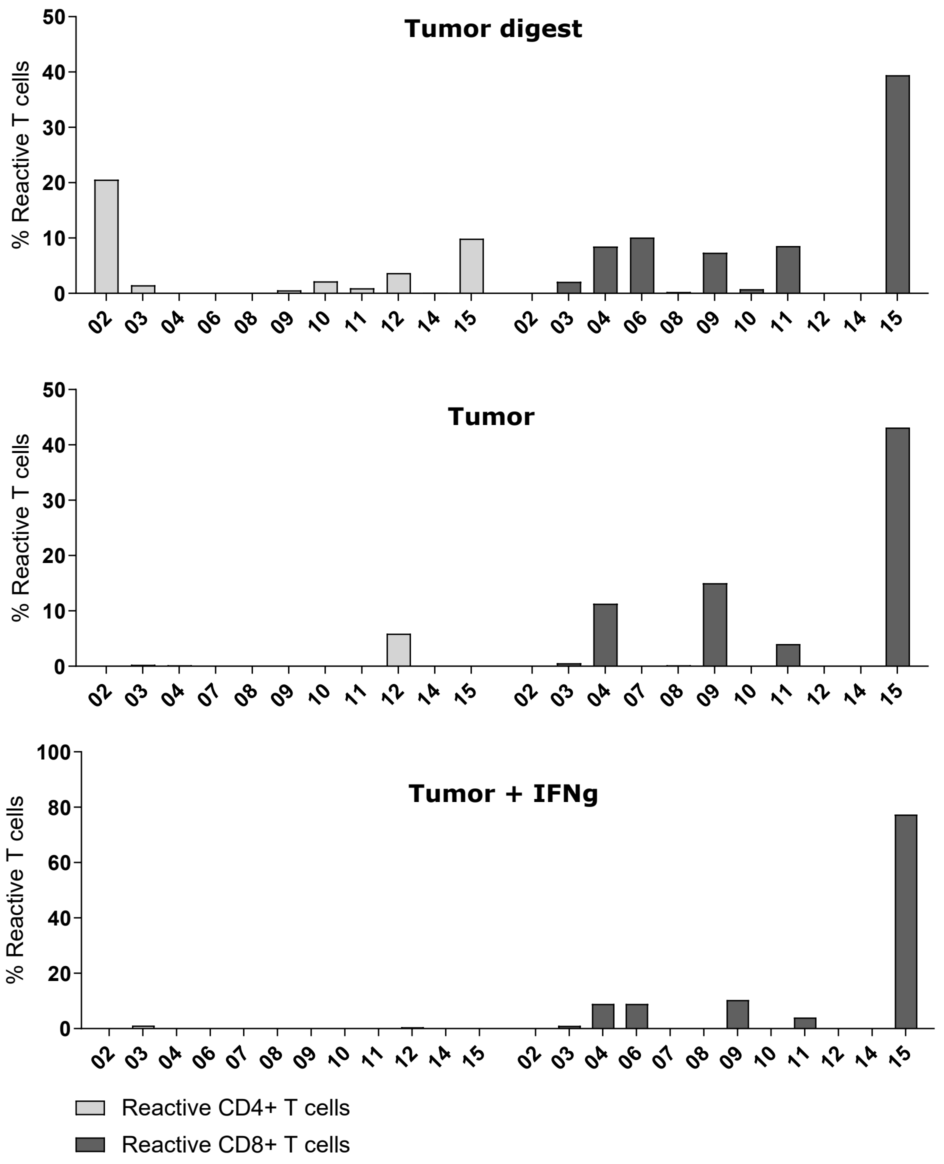

Figure S4: Assessment of tumor reactivity. *Infusion product reactivity towards autologous tumor digest, tumor cells and tumor cells pre-treated with IFNg was performed using multicytokine intracellular staining. Reactive cells were simultaneously positive to two out of the four reactivity markers used (CD137, CD107a, TNF, IFNg). Related to Figure 2.*

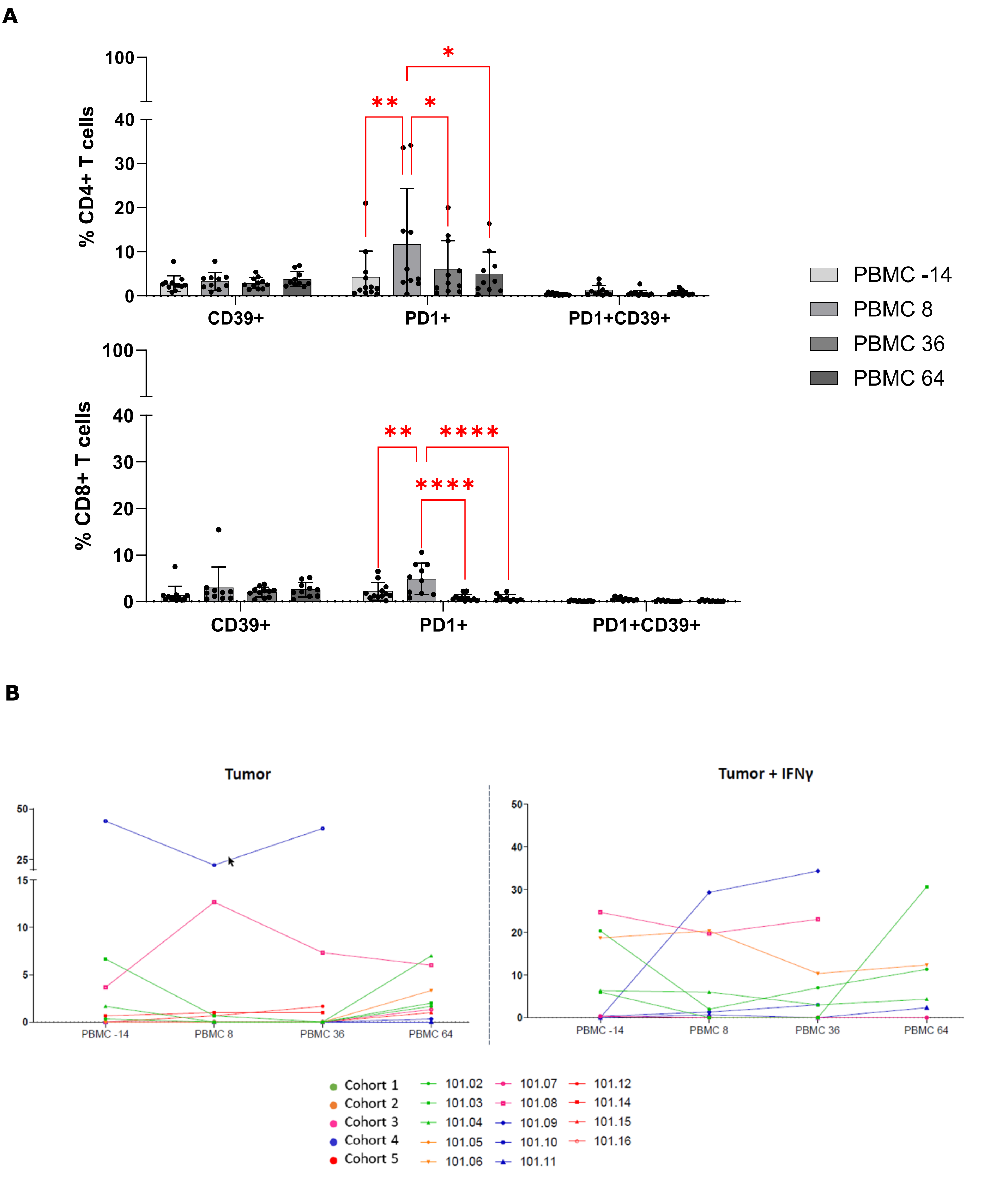

Figure S5: Phenotype and reactivity of PBMCs. ***(A)*** *Phenotypic characterization of PBMCS isolated from whole blood at different timepoints of the study. Significantly higher expression of PD1 on day 8 was observed in both peripheral CD4+ and CD8+ T cells populations. PBMCs phenotyping was performed on samples from patients treated at the Danish site (n=13). Data are presented as mean ± standard error of the mean (SEM). Statistical significance was evaluated using a two-way ANOVA followed by Tukey's multiple comparison test and indicated as p* <0.05, p** <0.01, p***<0.001 and p****<0.0001.* ***(B)*** *PBMCs reactivity towards tumor cells (with or without IFNg pre-treatment) was assessed using ex vivo IFNg ELISpot. Related to STAR*METHOD.*

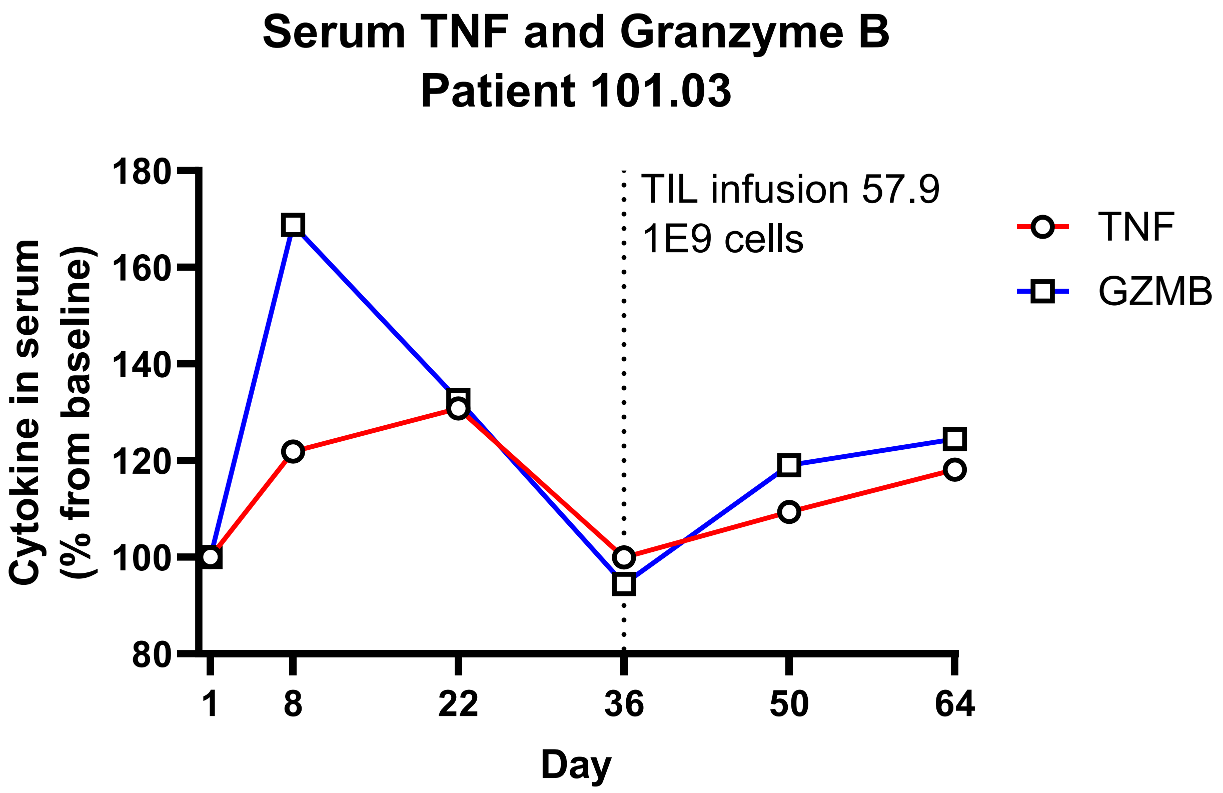

Figure S6: Pre-treatment serum cytokine levels of patient 101-03 on days 1, 8, 22, 36, 50 and 64. Related to STAR*METHOD

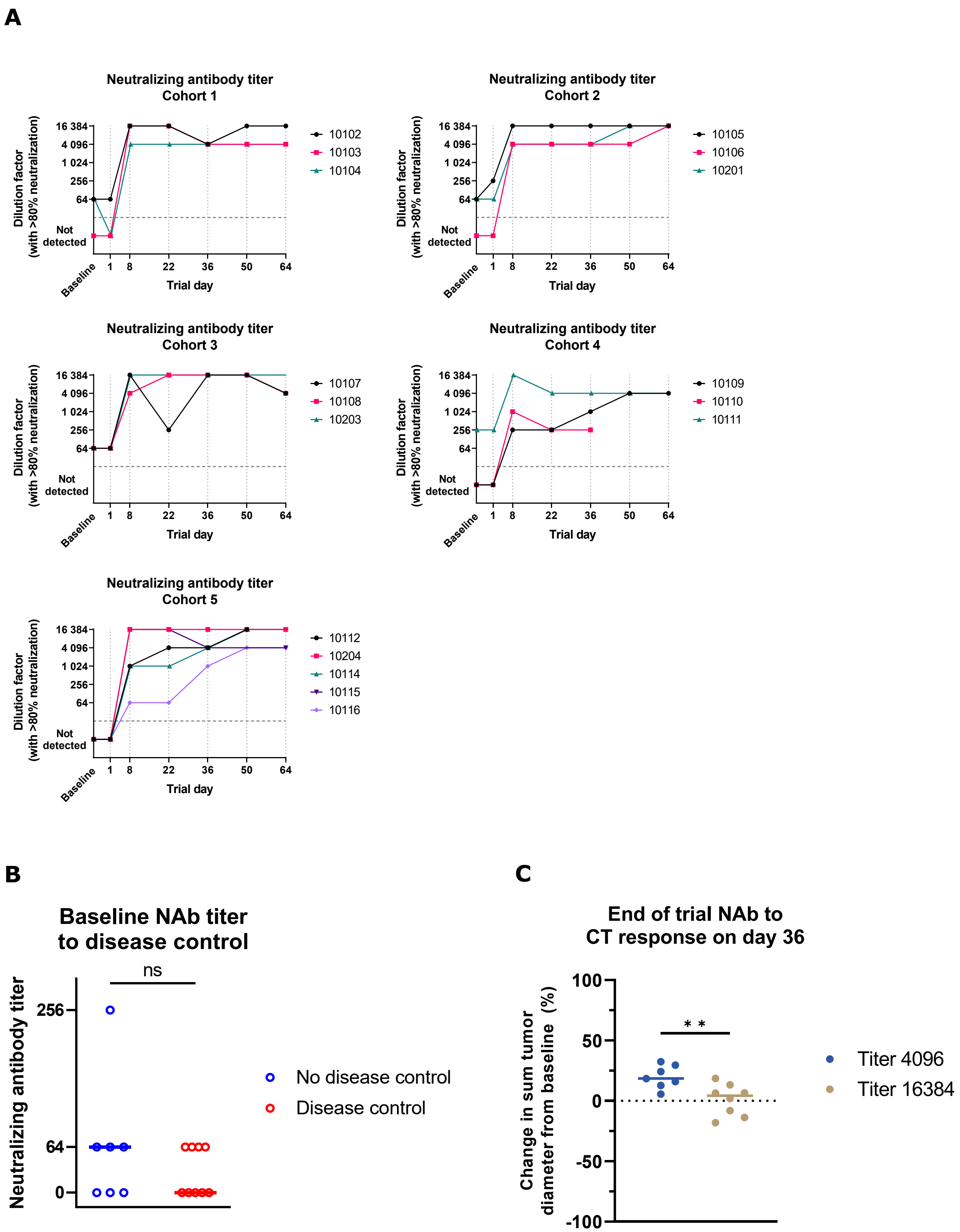

Figure S7: NAB titer measurement*. **(A)** Neutralizing antibody titer against TILT-123 across trial in all cohorts. **(B)** Baseline neutralizing antibody titer against TILT-123 in patients with or without disease control assessed with RECIST 1.1. at the end of the trial, n=16. ns=non-significant **(C)** End of trial neutralizing antibody titer against CT response on day 36. Groups compared with Mann-Whitney U-test. The mean value is indicated by the horizontal line. Statistical significance is indicated as p** <0.01. Related to STAR*METHOD*

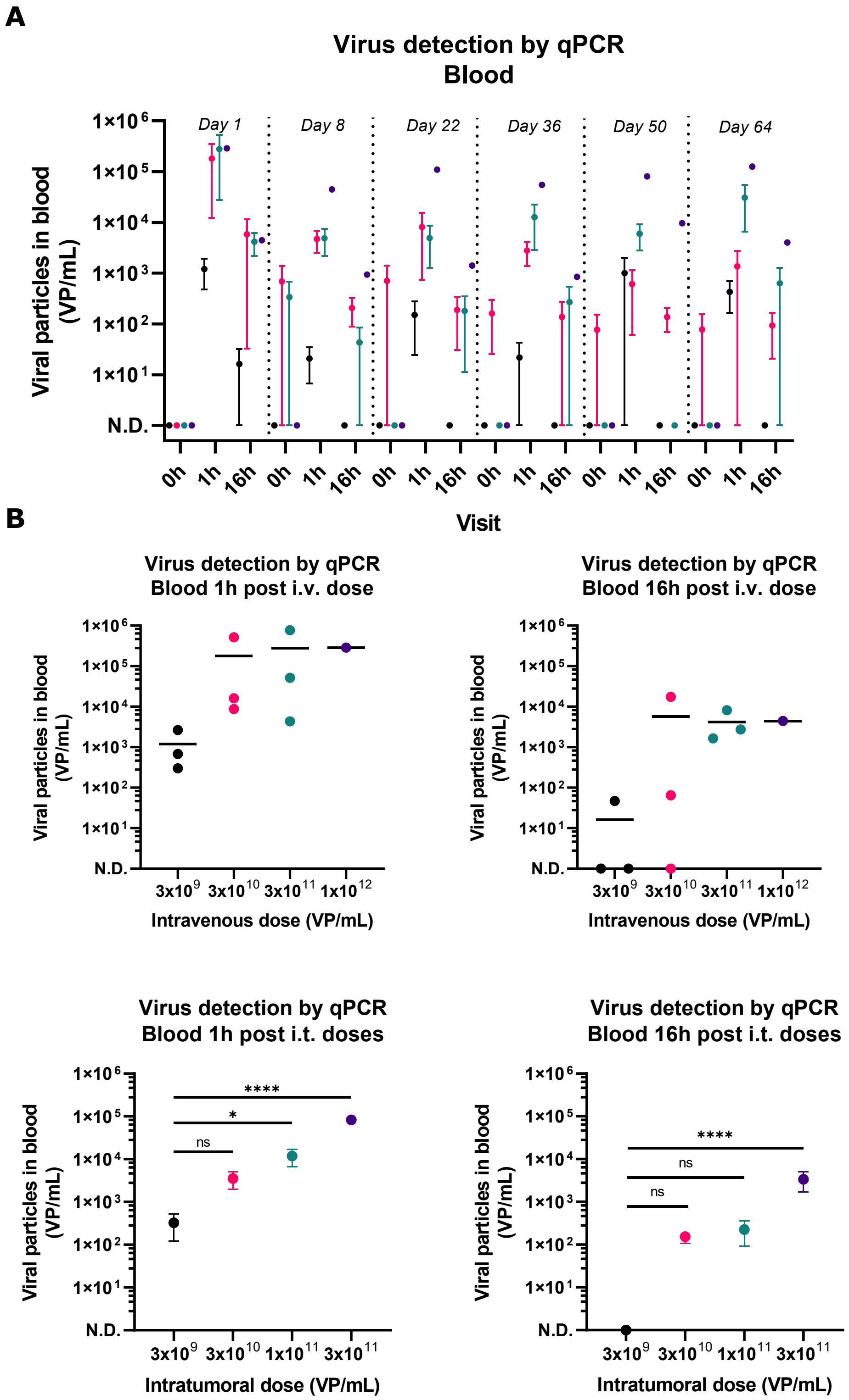

Figure S8: Virus genome detection in blood. (A) *Virus genome detection from blood across trial from cohorts 1-4.* ***(B)*** *Virus genome detection from blood 1 hour and 16 hours after intravenous (i.v.) and intratumoral (i.t.) doses. Groups were compared using two-way ANOVA followed by Fisher's Least Significant Difference (LSD) test. Mean values are indicated with horizontal lines for i.v. dose groups. Data is presented as mean* ± standard error of the mean (*SEM*) for i.t. dose groups. *Statistical significance is indicated as p* <0.05 and p****<0.0001. ns= non-significant. Related to STAR*METHOD.*

Patient	Localization of tumor resected for TIL expansion	Number of cells in TIL infusion product (x 10^9)	Localization of injected tumor(s)
**101.02**	Adrenal gland	36.8	Liver metastases
**101.03**	Subcutaneous	57.9	Subcutaneous tumors on leg
**101.04**	Lymph node	65.4	Tumors in left axillar
**101.05**	Liver	Na	Liver metastases
**101.06**	Subcutaneous	86.8	Liver metastases
**101.07**	Lymph node	69.2	Lymph node on neck
**101.08**	Subcutaneous	47.6	Lymph nodes on thoracic wall and neck
**101.09**	Subcutaneous	62.4	Liver tumor and subcutaneous tumor
**101.10**	Lymph node	63.2	Subcutaneous tumor on thoracic wall
**101.11**	Lymph node	17.9	Lymph nodes/inguinal tumors
**101.12**	Intraabdominal	28.7	Liver metastases
**101.14**	Lung	32.7	Liver metastases
**101.15**	Lymph node	44	Subcutaneous tumors on leg, Lymph node in inguen
**101.16**	Subcutaneous	48.9	Subcutaneous tumors on breast
**102.01**	Lymph node	2.58 + 4.9	Palate tumor<br>Tumor under right scapula<br>Tumors on left thigh and right thigh
**102.03**	Subcutaneous	3.51	Right axillary tumor, subcutaneous tumors on arm
**102.04**	Lymph node	3 + 2.1	Lymph node in inguen

Table S1: Treatment characteristics. *Localizations of resected tumors for TIL expansion, number of infused cells in the TIL product and localization of tumors injected with TIL-123. Related to STAR*METHOD.*

	Grade 1			Grade 2			Grade 3			Grade 4			Total	Unique cases
**Treatment Related Adverse Event**	**TILT-123**	**TIL**	**Both**	**TILT-123**	**TIL**	**Both**	**TILT-123**	**TIL**	**Both**	**TILT-123**	**TIL**	**Both**		
**General symptoms**														
Anorexia	1			1									2	2/17
Arthralgia			1										1	1/17
Asthenia	1		1	1									3	1/17
Dizziness	1												1	1/17
Fatigue	3	1	2										6	5/17
Fatigue worsening				1									1	2/17
Headache	3	1											4	3/17
Worsening of headache				3									3	1/17
Hypotension					1								1	1/17
Dry skin	1					1							2	2/17
Memory impairment			1										1	1/17
Flushing	1		1										1	2/17
Myalgia	3												3	2/17
Pain	1												1	1/17
Severe myodesopsia at rest	1												1	1/17
Maculopapular rash	2					1							2	3/17
**Local symptoms**														
Alopecia	2		1										3	3/17
Bilateral edema						1							1	1/17
Diarrhea	3												3	2/17
Edema, by the left eye	1												1	1/17
Lacrimation	2												2	1/17
Left thorax pain					1								1	1/17
Pain left knee	1												1	1/17
Pain left upper extremity	1												1	1/17
Pain liver			1										1	1/17
Pain right leg				1									1	1/17
Pruritus right crus	1												1	1/17
Muscle pain right calf	1												1	1/17
Skin redness right crus	1												1	1/17

Skin redness right groin	1												1	1/17
Swollen and tender lymph nodes right neck	1					1							2	1/17
**Infectious symptoms**														
Febrile syndrome		1											1	1/17
Fever	11	1	1	4	4	1	2	1					25	13/17
Chills	2		2		3	1		1					9	7/17
Abscess right side of thorax				1									1	1/17
**Injection related symptoms**														
Infection injection site				1									1	1/17
Itching at the injection sites	1												1	1/17
Injection site pain	3						1						4	3/17
**Hematology/lab values**														
Increased ALAT		1											1	1/17
Increased ASAT								1					1	1/17
Increased INR					1								1	1/17
Lymphopenia	1												1	1/17
TSH decreased	1												1	1/17
Prolonged QTcB interval			1										1	1/17
**Gastrointestinal**														
Nausea	4	2		2									8	6/17
Oral mucositis	1												1	1/17
Vomiting	2				1								3	2/17
**Other**														
Hypoxia					1								1	1/17
TIL reaction								1					1	1/17
Worsening of adrenal insuffciency											1			1/17
Vitiligo	2												2	2/17

Table S2: Adverse events. *Frequency and registered CTCAE grade of all treatment related adverse events according to CTCAE v.5.0. Related to Table 2.*

Patient	Gender	Histology	Treatment Cohort	Response day 36			BOR day 78 or later		
				RECIST 1.1	iRECIST	PET	RECIST 1.1	iRECIST	PET
**101.02**	Male	Mucosal	1	SD	iSD	SMD	PD	iUPD	PMD
**101.03**	Female	Cutaneous	1	PD	iUPD	PMD	SD	iSD	PMD
**101.04**	Male	Cutaneous	1	PD	iUPD	PMD	PD	iCPD	PMD
**101.05**	Male	Uveal	2	SD	iSD	NA	SD	iSD	NA
**101.06**	Male	Uveal	2	PD	iUPD	PMD	PD	iCPD	PMD
**102.01**	Female	Mucosal	2	PD	iUPD	SMD	PD	iCPD	PMD
**101.07**	Female	Mucosal	3	SD	iSD	PMR	CR*	iCR*	PCR*
**101.08**	Male	Cutaneous	3	PD	iUPD	PMD	PD	iCPD	PMD
**102.03**	Male	Cutaneous	3	PD	iUPD	SMD	PD	iCPD	SMD
**101.09**	Female	Uveal	4	PD	iUPD	SMD	PD	iCPD	SMD
**101.10**	Female	Mucosal	4	SD	iSD	MMR	SD	iSD	MMR
**101.11**	Female	Cutaneous	4	PD	iUPD	PMD	PD	iCPD	SMD
**101.12**	Female	Mucosal	5	PD	iUPD	PMD	NA	NA	NA
**101.14**	Male	Uveal	5	SD	iSD	PMR	SD	iSD	PMR
**101.15**	Female	Cutaneous	5	PD	iUPD	MMR	PR	iPR	PMR
**101.16**	Female	Cutaneous	5	PD	iUPD	SMD	PD	iCPD	PMD
**102.04**	Female	Cutaneous	5	SD	iSD	MMR	PD	iUPD	PMD

Table S3: Responses evaluated by RECIST 1.1, iRECIST and PET. *TILT-123 treatment alone was evaluated on day 36 before the infusion of TILs. TILT-123 and TIL treatment was evaluated as best overall response (BOR) on day 78 or later for patients continuing in the extension phase of the trial. * Pathological confirmed complete response. Patient 101.05 had PET-negative lesions at baseline. Patient 101.12 died from cancer progression before D78 evaluation. Related to Figure 2 and Figure 5.*

	Target	Fluorochrome	Clone	Species	specificity	Catalog number	Distributor
Golgi solution	CD107a	BV421	H4A3	mouse	anti-human	562623	BD
	GolgiStop™	Protein transport inhibitor containing Monensin				554724	BD
	GolgiPlug™	Protein transport inhibitor containing Brefeldin A				555029	BD
Extracellular staining ICS	CD4	BV711	SK3	mouse	anti-human	563028	BD
	CD8	QD605	3B5	mouse	anti-human	Q10009	Invitrogen
	CD56	BV510	NCAM16.2	mouse	anti-human	563041	BD
	CD3	PE-CF594	UCHT1	mouse	anti-human	562280	BD
Intracellular staining ICS	TNFα	APC	MAb11	mouse	anti-human	554514	BD
	IFN-γ	PeCy7	B27	mouse	anti-human	557643	BD
	CD137	PE	4B4-1	mouse	anti-human	555956	BD
Phenotyping panel 1	CD3	BV786	SK7	mouse	anti-human	563800	BD
	HLA-DR	PerCP-Cy5.5	G46-6	mouse	anti-human	560652	BD
	CCR7	PE	G043H7	mouse	anti-human	353204	Nordic biosite
	CD8	APC-R700	RPA-T8	mouse	anti-human	565165	BD
	CD39	BV421	TU66	mouse	anti-human	563679	BD
	CD4	BV510	SK3	mouse	anti-human	562970	BD
	CD56	BV605	NCAM16.2	mouse	anti-human	562780	BD
	CD45RA	BV650	HI100	mouse	anti-human	563963	BD
	PD-1	PE/Dazzle 594	EH12.2H7	mouse	anti-human	329940	Nordic biosite
	CD38	PE-CY5	HIT2	mouse	anti-human	555461	BD
Phenotyping panel 2	CD127	FITC	REA614	mouse	anti-human	130-113-417	Miltenyi Biotec
	CXCR3	BB700	1C6	mouse	anti-human	566532	BD
	CCR4	APC	L291H4	mouse	anti-human	359408	Nordic biosite
	CCR6	BV711	11A9	mouse	anti-human	563923	BD
	CD3	BV786	SK7	mouse	anti-human	563800	BD
	CD4	BV510	SK3	mouse	anti-human	562970	BD
	CD56	BV605	NCAM16.2	mouse	anti-human	562780	BD
	CD8	APC-R700	RPA-T8	mouse	anti-human	565165	BD
	CD25	PE-CY7	2A3	mouse	anti-human	335824	BD
Phenotyping panel 3	CD3	BV786	SK7	mouse	anti-human	563800	BD
	CD19	BV711	SJ25C1	mouse	anti-human	563036	BD
	HLA-DR	PerCP-Cy5.5	G46-6	mouse	anti-human	560652	BD
	CD1c	APC	L161	mouse	anti-human	331524	Nordic biosite
	CD33	BV510	WM53	mouse	anti-human	563257	BD
	CD123	BV605	7G3	mouse	anti-human	564197	BD
	CD11c	BV650	B-ly6	mouse	anti-human	563404	BD
	CD56	BV605	NCAM16.2	mouse	anti-human	562780	BD
	CD141	PE	1A4	mouse	anti-human	559781	BD
	CD16	PE/AF700	3G8	mouse	anti-human	101611P1	AAT Bioquest
	CD14	PE-CFE594	MφP9	mouse	anti-human	562335	BD

Table S4: List of antibodies used for multicytokine intracellular staining and phenotyping. *Related to STAR*METHOD*
